# Proteolysis‐Based Biomarker Repertoire of the Neurofilament Proteome

**DOI:** 10.1111/jnc.70023

**Published:** 2025-03-11

**Authors:** Axel Petzold

**Affiliations:** ^1^ Queen Square Institute of Neurology, UCL and The National Hospital for Neurology and Neurosurgery London UK

**Keywords:** Alzheimer disease, Boman index, Charcot‐MarieTooth disease, dementia, neurodegenerative disease, neurofilament biomarker

## Abstract

Neurodegeneration presents a significant challenge in ageing populations, often being detected too late for effective intervention. Biomarkers have shown great potential in addressing this issue, with neurofilament (Nf) proteins emerging as validated biomarkers presently transitioning from research to routine laboratory use. Whilst advances in large‐scale quantitative analyses have enabled the targeted study of proteolytic Nf fragments in blood, the complete landscape of the Nf proteolytic breakdown remains unknown. This study presents a comprehensive atlas of the human Nf isoform (*Z*) degradome, based on the number of known cleavage sites (*x*). The full scale of the Nf degradome is described by the formula: *Z* = ((*x* + 1) × (*x* + 2)/2) − 1. The resulting neurofilament degradome atlas (NDA) was validated through a triple‐layer approach using in vitro data (open access at: https://doi.org/10.5522/04/25689378.v1). The NDA offers valuable applications in biomarker detection, targeted antibody development, exploration of autoimmunity and understanding Nf aggregate formation. Analysis of the Nf degradome reveals novel insights into neurodegenerative diseases by investigating peptide pools affected by genetic mutations in the Nf genome and alterations in proteolytic pathways. The annotated NDA is publicly available as a database resource, supporting advancements in affinity‐based biomarker tests through informed peptide selection and minimising biases in label‐free approaches. In conclusion, this study highlights the biological significance of a dynamic pool of coexisting proteolytic Nf peptides, providing a framework that can be applied to other proteins.

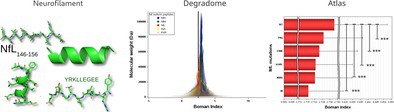

## Graphical abstract

Protein degradation underlies essential biological processes, and its dysfunction can contribute to disease. By systematically charting the neurofilament degradome, this study offers a conceptual framework and methodological blueprint for mapping a complete protein degradome atlas. Through combinatorial analysis of known cleavage sites, the resulting peptide products were aligned to the annotated proteome, revealing numerous biomarker candidates. A triple‐validation approach confirmed these matches, emphasising the atlas's potential for uncovering nuanced biomarker signatures in paleoproteomics, human disease, genetic mutations, cell biology and protein aggregation. This research paves the way for novel, highly sensitive and specific biomarker discoveries.

**Abbreviations**
ACN: acetonitrile
ALS: amyotrophic lateral sclerosis
ANuPP: aggregation nucleation prediction in peptides and proteins
APR: amyloidogenic properties
Arg: arginine
Asp: aspartate
CMT: Charcot‐Marie‐Tooth disease
CNS: central nervous system
CSF: cerebrospinal fluid
Cys: cysteine
Da: Dalton
EDTA: ethylene‐diamine‐tetra‐acetic disodium salt
Glu: glutamine
HRP: horseradish peroxidase
HSP: hereditary spastic paraplegia
ID: identifier
INA: internexin alpha
iPSC: induced pluripotent stem cells
kDa: kilo Dalton
lDDT: local distance difference test
LDS: lithium dodecyl sulfate
Lys: lysine
MS: mass spectrometer
Mw: molecular weight
NDA: neurofilament degradome atlas
Nf: neurofilament
NfH: neurofilament heavy chain
NfL: neurofilament light chain
NfM: neurofilament medium chain
PD: Parkinson disease
pH: potential of hydrogen
pI: basal isoelectric point
PNS: peripheral nervous system
PRIDE: PRoteomics IDEntifications
Pro: proline
PRP: peripherin
PTM: posttranslational modifications
RPM: revolutions per minute
SELDI: surface‐enhanced laser desorption/ionisation
TOF: time‐of‐flight

## 1 Introduction

Building on neurofilament (Nf) proteins proven role as a reliable biomarker for neurodegeneration (Khalil et al. 2018, 2024), a 10‐item agenda for Nf research has been proposed (Petzold 2022). The first four items on this list focus on proteolysis, expanding the repertoire of Nf isoforms, specificity and sensitivity, and the relationship between Nf aggregation and disease association (Petzold 2022). This study addresses these four priorities systematically, beginning with proteolysis.

The breakthrough ability to quantify Nf from blood samples is thought to be the consequence of proteolysis (Khalil et al. 2024; De Paoli et al. 2024). However, the scope of proteolytic breakdown products of Nf is not known. Current affinity‐based tests are biassed to antibodies used (De Paoli et al. 2024; Shaw et al. 2023; Khalil et al. 2024). This remains an important limitation to expanding from affinity‐based quantitative methods to novel label‐free approaches (Leckey et al. 2024).

**FIGURE 1:**
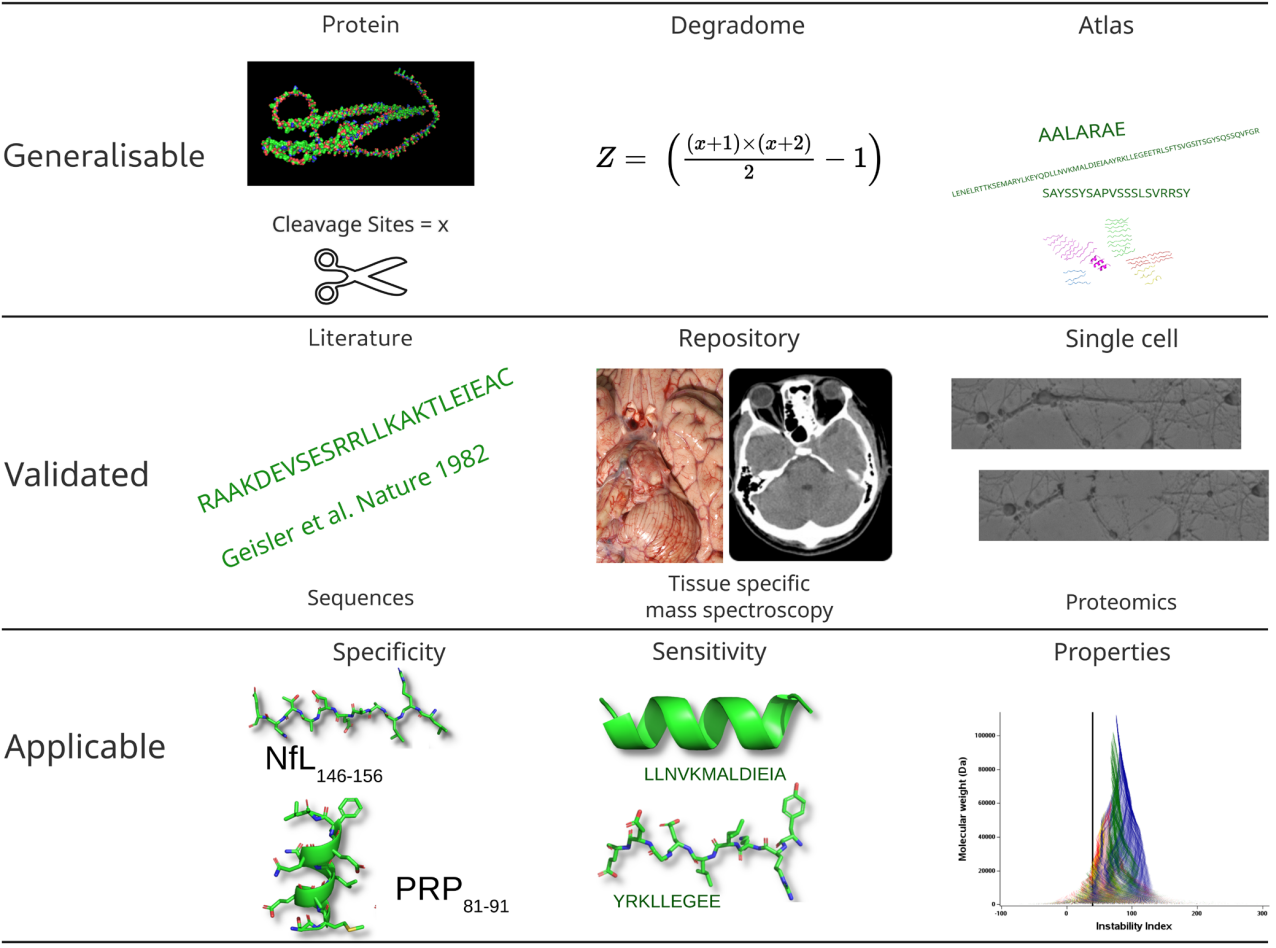
Introduction to the multi‐step process used to generate and validate the NDA. Nf isoform sequences and cleavage sites: Five Nf isoform sequences were cleaved at all currently known cleavage sites (*x*), yielding a comprehensive Nf Degradome with *Z* peptides. These extracted peptide sequences formed the foundation for the NDA. Alignment with SwissProtein Database: The resulting peptide sequences were then aligned with the SwissProtein database. This step allowed labelling of each sequence as unique to a specific Nf isoform, shared among multiple Nf isoforms, or shared with other proteins. The annotated NDA underwent three independent validation steps: (i) Literature Review: Cross‐referencing against published data on Nf cleavage products. (ii) Match with Repositories: Comparing sequences to public data repositories (e.g., PRIDE). (iii) Single‐cell proteomics: Confirming the presence of low‐abundance Nf sequences at the individual‐cell level. Finally, the applications of the NDA entailed: Labelling of sequences, comprehensive peptide property data, and insights into diseases. Peptides were categorized according to whether they were unique (specificity, sensitivity) or shared across Nf isoforms, the human proteome, or other species' proteomes. In addition, the NDA includes key protein features, such as aggregation potential, nucleation predictions, disease associations (genetic/acquired), and targets potentially relevant to drug development. Analysing these properties at the peptide‐pool level, rather than focusing only on full‐length Nf isoforms, provides novel insights into neurodegenerative disease biology.

Therefore, this paper seeks to bridge this gap through an approach that is systematic, generalisable, and has direct application potential (Figure 1). A comprehensive dataset is amassed for the Nf heavy (NfH), medium (NfM), light (NfL) chains, *α*‐internexin (INA) and peripherin (PRP). The dataset is matched with the UniProtKB/SwissProtein database. Statistical analyses combined with a triple‐validation strategy are used to extract key peptides, which are then systematically organised and investigated for biochemical features with a focus on the likelihood of protein aggregation. Peptide pool profile patterns emerging are interrogated regarding their associations with diseases ranging from genetics to autoimmunity.

The results of the present study suggest shifting the focus from individual, full‐length Nf proteins (Khalil et al. 2024; Leckey et al. 2024; De Paoli et al. 2024) to a biologically more realistic scenario: a dynamic pool of coexisting proteolytic peptides.

## 2 Methods

### 2.1 Ethics Approval and Informed Consent Statement

Ethical approval for this study was given (centre number 875KLH, study number 03/N101, UK). This study was conducted in accordance with the UK Human Tissue Act 2004. Informed consent was obtained.

### 2.2 Antibodies

Mouse monoclonal IgG1 antibodies were obtained from different suppliers: Abcam, ThermoFisher, Sigma‐Aldrich, and Sternberger Monoclonals (now Covance Research Products). The antibodies used in the study were as follows: anti‐NfH (SMI32, SMI33, SMI34, SMI35, SMI37, SMI38, SMI310 (The original catalogue number refers to the clone from Sternberger Monoclonals Incorporated (SMI). The complete original catalogue can be requested from the author)), anti‐NfM (Abcam Cat# ab7794, RRID:AB_306083, clone NF‐09), anti‐NfL (Sigma‐Aldrich, Cat# N5139, RRID:AB_477276, clone NR4), anti‐INA (ThermoFisher, Cat# 32‐3600, clone 2E3), peripherin (Sigma‐Aldrich, Cat# P5117, RRID:AB_477360, clone P5117), and anti‐glial fibrillary acidic protein (SMI26). It should be noted that SMI26 is a cocktail of the Bigner‐Eng clones MAb2E1, Mab1B4, MAb2E1, Mab4A11, and IgG from SMI21. The choice of antibodies targeting specific Nf phosphorylation states was guided by prior immunoblotting and immunohistochemical studies, as previously summarized (Petzold et al. 2003; Petzold 2005). Horseradish peroxidase (HRP)‐conjugated rabbit anti‐mouse IgG was purchased from Dako (now Agilent, Cat# P026002‐2).

### 2.3 Chemicals

Analytical‐grade NaCl, ethylene‐diamine‐tetra‐acetic disodium salt (EDTA), sodium barbitone, barbitone, Tween 20 and methanol were obtained from Sigma. The following products from Novex (now ThermoFisher) were used: NuPage Electrophoresis System, Lithium Dodecyl Sulfate (LDS), NOVEX 3%–8% Tris‐Acetate 2D gels (Cat# EA0376), NOVEX MOPS buffer (Cat# NP0001), and NOVEX transfer buffer (Cat# NP0006). All buffers and solutions were prepared according to the instructions provided by the suppliers. Sinapinic acid was purchased from Ciphergen (Surrey Research Park, Guilford, Surrey, UK). The chemiluminescence substrate used was Pierce SuperSignal Chemiluminescent Substrate (Luminal/Enhancer, Component No. 34080, No. 1856136; Stable Peroxide Solution, Component No. 34080; No. 1856135) purchased from Pierce (Rockford, Illinois 61105, USA).

The preparation of buffers and solutions involved the following steps: Barbitone buffer was prepared by dissolving 13.1 g of sodium barbitone, 2.1 g of barbitone, and 465 mg of EDTA. The washing solution consisted of 0.1% dry skimmed milk in a barbitone buffer with 0.025% Tween 20. The blocking solution was prepared by dissolving 2% dry skimmed milk in a barbitone buffer. For antibody dilution, 0.1% dry skimmed milk in a barbitone buffer was used.

### 2.4 Samples

Auto‐proteolysis of the spinal cord tissue (*n* = 1) was allowed to occur for 48 h at 37°C (lot 1) and 5 months at 4°C (lot 2), after which the spinal cord tissue was processed. This choice on timing was made because previous data showed a time‐dependent pattern of Nf iso‐ and phosphoform degradation (Goldstein et al. 1987; Petzold et al. 2020; Shaw et al. 2023). Briefly, the tissue was homogenised with ultrasound and dissolved in 700 μL of barbitone buffer. The resulting spinal cord homogenate was subjected to ultra‐centrifugation at 140 000 RPM for 10 min. The supernatant was then collected and stored in aliquots at −80°C until further analysis.

PC6‐3 cells were cultured and induced to adopt a neuronal‐like phenotype as described (Pittman et al. 1993). After induction, the cells were transferred onto polyethylene naphthalate membrane–coated glass slides for downstream analyses. They were then washed three times with PBS to remove any residual medium. Following these washes, the samples were either fixed in 70% ethanol or allowed to undergo auto‐proteolysis until air‐dried, enabling controlled observation of proteolytic processes whilst maintaining cell morphology.

### 2.5 Immunoblotting

Two auto‐preolysed spinal cord homogenate samples (incubated for 48 h and 5 months, respectively) were each mixed with LDS and heated at 65°C for 10 min. A 700 μL volume of each sample was then loaded into the large well of a 2D gel. Electrophoresis was carried out at 150 V and 45 mA for 1.5 h using MOPS running buffer.

Following electrophoresis, the gel was trimmed and transferred onto a Hybond‐P membrane in a NOVEX transfer chamber for 1 h (25 V, 100 mA). The membrane was then incubated for 1 h in blocking solution on a slow rocker. Next, it was placed in a dry polypropylene incubation chamber containing 25 mL of washing solution. After decanting the washing solution, primary antibodies (1:1000 in antibody diluent) were added, and the chamber was sealed and kept on a slow rocker at 4°C overnight.

On the following day, the membrane was washed six times for 10 min each in washing solution, then incubated with HRP‐labelled secondary antibodies (1:2500 in antibody diluent) for 1 h. After another six washes, the membrane was developed using a chemiluminescent substrate and exposed onto photographic paper in a dark room.

### 2.6 Surface‐Enhanced Laser Desorption/Ionisation

#### 2.6.1 SELDI‐TOF‐MS Procedure

Surface‐enhanced laser desorption/ionisation (SELDI) analysis utilized Ciphergen's reactive‐surface protein chip arrays, with measurements conducted on a linear time‐of‐flight (TOF) mass spectrometer (MS) stationed at Surrey Research Park, Guildford, Surrey, UK. The SELDI‐TOF‐MS apparatus operated in a positive ion mode, configured with a source voltage of 20,000 V, a pulse‐time lag fixed at 1963 ns, and a deflector mass cutoff set to 10 000 Da (Mills et al. 2006).

#### 2.6.2 Sample Preparation and Binding

The primary binding buffer, Barbitone‐EDTA at pH 9.6, was used, allocating 150 μL per spot on the anion‐exchange Q10 chip. For specimens rich in neuronal cells, the Q10 chip was directly incubated with 100 μL of the sample within a fully assembled bioprocessor unit. In samples with fewer neuronal cells (< 100 cells), a laser capture microscope was employed for precise cell targeting and deposition onto the chip's binding surface. This was followed by an hour‐long incubation with vigorous shaking. Post‐incubation, each chip was subjected to two wash cycles, each using 150 μL of the Barbitone‐EDTA buffer and including a 5‐min incubation.

#### 2.6.3 Chip Analysis Preparation

Prior to analysis, each chip was primed with 150 μL of H_2_O, agitated briefly for 10 s, and then left to air dry for 2 h at ambient temperature. The matrix for detection, a mixture of sinapinic acid (5 mg), 125 μL of acetonitrile (ACN), and 0.1% trifluoroacetic acid (TFA), was freshly prepared. I applied 1 μL of this matrix solution to each chip spot twice, allowing them to air dry before proceeding. The final SELDI‐TOF‐MS examination was executed using a laser intensity of 200 and a sensitivity level adjusted to 4.

### 2.7 Laser Capture Microscopy

The Laser Microbeam System (P.A.L.M., Microlaser Technologies AG, Munich, Germany) was employed with visual guidance for the capture of neurons in barbitone buffer (pH 8.9) intended for SELDI‐TOF‐MS analysis. Importantly, for single‐cell proteomic experiments, the neurons were directly captured onto the chip surface in order to avoid losing peptides through non‐specific binding to laboratory material surfaces during sample handling.

### 2.8 Statistics

All statistical analyses and graphs were prepared using BLAST+ and SAS software (version 9.4 m7, SAS Institute Inc., Cary, North Carolina, USA). Descriptive statistics were performed graphically in the form of scatter plots and histograms and statistically using the Shapiro–Wilk test. The paired *T*‐test was used for the comparison of two variables with a normal, Gaussian distribution. The mean and standard deviation were reported. Independent categorical variables with one quantitative dependent variable were analysed by the one‐way ANOVA test. For the comparison of more than two variables, generalised linear models (GLM) were used, showing the *F*‐value and the exact *p*‐values. All graphs were prepared with *proc sgplot* on data from the neurofilament degradome atlas (NDA) using *histogram, scatter, reg, heatmap* and *proc sgpie*. Because of the large amount of data, there is a need to manually increase the maximum number of observations and bins available to SAS in the *ods graphics* declaration by including *obsmax* = *100000000* and *NXYBINSMAX* = *10000000*. Likewise, the maximal number of slices for the pie charts was set by hand to *MAXSLICES* = *100 000* and dognoutsize to *ringsize* = *0.5*. For density scatter plots with large data, the option *transparency* = *0.95* was added to *proc scatter*.

For the compilation of the NDA, SAS was initially used for the identification of 100% identical peptide sequences between the Nf isoforms because of processing speed and because BLAST+ was not designed for this purpose. Next, BLAST+ was used for pairwise alignment for the identification of epitope matches. Each output from BLAST+ was imported to SAS to check for duplicates using *proc sort nodupkey* and assessment of field integrity. Outputs of the final NDA were exported from SAS using *data [*…*] run*; tailored at *fasta* files with *put* “>” *NDA_ID*; *put peptide*; or for .*csv* with *proc export*.

No formal outlier test was conducted on the data, and no data points were removed.

### 2.9 Nf Polypeptides Calculations

The full protein sequences were downloaded from UniProtKB/Swiss‐Prot using the identifier P07196 for NFLHUMAN, P07197 for NFMHUMAN, P12036 for NFHHUMAN, Q16352 for AINXHUMAN (*α*‐internexin) and P41219 for peripherin (PRP). The full protein sequences (see Table 1) were processed by PeptideCutter for identification of all known cleavage sites (Assessed on 11‐JUL‐2021 and 30‐DEC‐2022. The number of cleavage sides remained the same on both dates). Let *x* be the number of cleavage sites in a protein. The total number of possible polypeptides for this protein (*Z*) is calculated by: $Z=\frac{\left(x+1\right)\times \left(x+2\right)}{2}-1$.

**TABLE 1:** Neurofilament isoform amino acid sequences.

Nf isoform	Amino acid sequence
NfH	MMSFGGADALLGAPFAPLHGGGSLHYALARKGGAGGTRSAAGSSSGFHSWTRTSVSSVSASPSRFRGAGAASSTDSLDTLSNGPEGCMVAVATSRSEKEQLQALNDRFAGYIDKVRQLEAHNRSLEGEAAALRQQQAGRSAMGELYEREVREMRGAVLRLGAARGQLRLEQEHLLEDIAHVRQRLDDEARQREEAEAAARALARFAQEAEAARVDLQKKAQALQEECGYLRRHHQEEVGELLGQIQGSGAAQAQMQAETRDALKCDVTSALREIRAQLEGHAVQSTLQSEEWFRVRLDRLSEAAKVNTDAMRSAQEEITEYRRQLQARTTELEALKSTKDSLERQRSELEDRHQADIASYQEAIQQLDAELRNTKWEMAAQLREYQDLLNVKMALDIEIAAYRKLLEGEECRIGFGPIPFSLPEGLPKIPSVSTHIKVKSEEKIKVVEKSEKETVIVEEQTEETQVTEEVTEEEEKEAKEEEGKEEEGGEEEEAEGGEEETKSPPAEEAASPEKEAKSPVKEEAKSPAEAKSPEKEEAKSPAEVKSPEKAKSPAKEEAKSPPEAKSPEKEEAKSPAEVKSPEKAKSPAKEEAKSPAEAKSPEKAKSPVKEEAKSPAEAKSPVKEEAKSPAEVKSPEKAKSPTKEEAKSPEKAKSPEKAKSPEKEEAKSPEKAKSPVKAEAKSPEKAKSPVKAEAKSPEKAKSPVKEEAKSPEKAKSPVKEEAKSPEKAKSPVKEEAKTPEKAKSPVKEEAKSPEKAKSPEKAKTLDVKSPEAKTPAKEEARSPADKFPEKAKSPVKEEVKSPEKAKSPLKEDAKAPEKEIPKKEEVKSPVKEEEKPQEVKVKEPPKKAEEEKAPATPKTEEKKDSKKEEAPKKEAPKPKVEEKKEPAVEKPKESKVEAKKEEAEDKKKVPTPEKEAPAKVEVKEDAKPKEKTEVAKKEPDDAKAKEPSKPAEKKEAAPEKKDTKEEKAKKPEEKPKTEAKAKEDDKTLSKEPSKPKAEKAEKSSSTDQKDSKPPEKATEDKAAKGK
NfM	MSYTLDSLGNPSAYRRVTETRSSFSRVSGSPSSGFRSQSWSRGSPSTVSSSYKRSMLAPRLAYSSAMLSSAESSLDFSQSSSLLNGGSGPGGDYKLSRSNEKEQLQGLNDRFAGYIEKVHYLEQQNKEIEAEIQALRQKQASHAQLGDAYDQEIRELRATLEMVNHEKAQVQLDSDHLEEDIHRLKERFEEEARLRDDTEAAIRALRKDIEEASLVKVELDKKVQSLQDEVAFLRSNHEEEVADLLAQIQASHITVERKDYLKTDISTALKEIRSQLESHSDQNMHQAEEWF KCRYAKLTEAAEQNKEAIRSAKEEIAEYRRQLQSKSIELESVRGTKESLERQLSDIEERHNHDLSSYQDTIQQLENELRGTKWEMARHLREYQDLLNVKMALDIEIAAYRKLLEGEETRFSTFAGSITGPLYTHRPPITISSKIQKPKVEAPKLKVQHKFVEEIIEETKVEDEKSEMEEALTAITEELAVSMKEEKKEAAEEKEEEPEAEEEEVAAKKSPVKATAPEVKEEEGEKEEEEGQEEEEEEDEGAKSDQAEEGGSEKEGSSEKEEGEQEEGETEAEAEGEEAEAKEEKKVEEKSEEVATKEELVADAKVEKPEKAKSPVPKSPVEEKGKSPVPKSPVEEKGKSPVPKSPVEEKGKSPVPKSPVEEKGKSPVSKSPVEEKAKSPVPKSPVEEAKSKAEVGKGEQKEEEEKEVKEAPKEEKVEKKEEKPKDVPEKKKAESPVKEEAVAEVVTITKSVKVHLEKETKEEGKPLQQEKEKEKAGGEGGSEEEGSDKGAKGSRKEDIAVNGEVEGKEEVEQETKEKGSGREEEKGVVTNGLDLSPADEKKGGDKSEEKVVVTKTVEKITSEGGDGATKYITKSVTVTQKVEEHEETFEEKLVSTKKVEKVTSHAIVKEVTQSD
NfL	MSSFSYEPYYSTSYKRRYVETPRVHISSVRSGYSTARSAYSSYSAPVSSSLSVRRSYSSSSGSLMPSLENLDLSQVAAISNDLKSIRTQEKAQLQDLNDRFASFIERVHELEQQNKVLEAELLVLRQKHSEPSRFRALYEQEIRDLRLAAEDATNEKQALQGEREGLEETLRNLQARYEEEVLSREDAEGRLMEARKGADEAALARAELEKRIDSLMDEISFLKKVHEEEIAELQAQIQYAQISVEMDVTKPDLSAALKDIRAQYEKLAAKNMQNAEEWF KSRFTVLTESAAKNTDAVRAAKDEVSESRRLLKAKTLEIEACRGMNEALEKQLQELEDKQNADISAMQDTINKLENELRTTKSEMARYLKEYQDLLNVKMALDIEIAAYRKLLEGEETRLSFTSVGSITSGYSQSSQVFGRSAYGGLQTSSYLMSTRSFPSYYTSHVQEEQIEVEETIEAAKAEEAKDEPPSEGEAEEEEKDKEEAEEEEAAEEEEAAKEESEEAKEEEEGGEGEEGEETKEAEEEEKKVEGAGEEQAAKKKD
INA	MSFGSEHYLCSSSSYRKVFGDGSRLSARLSGAGGAGGFRSQSLSRSNVASSAACSSASSLGLGLAYRRPPASDGLDLSQAAARTNEYKIIRTNEKEQLQGLNDRFAVFIEKVHQLETQNRALEAELAALRQRHAEPSRVGELFQRELRDLRAQLEEASSARSQALLERDGLAEEVQRLRARCEEESRGREGAERALKAQQRDVDGATLARLDLEKKVESLLDELAFVRQVHDEEVAELLATLQASSQAAAEVDVTVAKPDLTSALREIRAQYESLAAKNLQSAEEWYKSKFANLNEQAARSTEAIRASREEIHEYRRQLQARTIEIEGLRGANESLERQILELEERHSAEVAGYQDSIGQLENDLRNTKSEMARHLREYQDLLNVKMALDIEIAAYRKLLEGEETRFSTSGLSISGLNPLPNPSYLLPPRILSATTSKVSSTGLSLKKEEEEEEASKVASKKTSQIGESFEEILEETVISTKKTEKSNIEETTISSQKI
PRP	MSHHPSGLRAGFSSTSYRRTFGPPPSLSPGAFSYSSSSRFSSSRLLGSASPSSSVRLGSFRSPRAGAGALLRLPSERLDFSMAEALNQEFLATRSNEKQELQELNDRFANFIEKVRFLEQQNAALRGELSQARGQEPARADQLCQQELRELRRELELLGRERDRVQVERDGLAEDLAALKQRLEEETRKREDAEHNLVLFRKDVDDATLSRLELERKIESLMDEIEFLKKLHEEELRDLQVSVESQQVQQVEVEATVKPELTAALRDIRAQYESIAAKNLQEAEEWYKSKYADLSDAANRNHEALRQAKQEMNESRRQIQSLTCEVDGLRGTNEALLRQLRELEEQFALEAGGYQAGAARLEEELRQLKEEMARHLREYQELLNVKMALDIEIATYRKLLEGEESRISVPVHSFASLNIKTTVPEVEPPQDSHSRKTVLIKTIETRNGEVVTESQKEQRSELDKSSAHSY

*Note:* Proteolytic cleavage sites are colour coded in magenta if targeted by 8 different enzymes, yellow for 7 enzymes and green for 6 enzymes. For identification of Nf cleavage sites subject to five or less enzymes see Table 2.

Enzymes and chemicals used for Nf isoform cleavage products in PeptideCutter: Arg‐C proteinase, Asp‐N endopeptidase, Asp‐N endopeptidase + N‐terminal Glu, BNPS‐Skatole, Caspase1, Caspase2, Caspase3, Caspase4, Caspase5, Caspase6, Caspase7, Caspase8, Caspase9, Caspase10, Chymotrypsin‐high specificity (C‐term to [FYW], not before P), Chymotrypsin‐low specificity (C‐term to [FYWML], not before P), Clostripain, CNBr, Enterokinase, Granzyme B, Factor Xa, Formic acid, Glutamyl endopeptidase, Hydroxylamine, Iodosobenzoic acid, LysC, LysN, NTCB (2‐nitro‐5‐thiocyanobenzoic acid), Pepsin (at pH 1.3 & pH < 2), Proline‐endopeptidase, Proteinase K, Staphylococcal peptidase I, Tobacco etch virus protease, Thermolysin, Thrombin and Trypsin (SIB Swiss Institute of Bioinformatics 2021).

### 2.10 In Silico Proteolysis

Coding was done in Python to produce a list of all cleavage products for Nf isoforms from the FASTA data format. The coded algorithms are available freely for download from figshare.

### 2.11 BLAST

To assess the level of amino acid sequence similarities, the Nf isoforms were compared to each other and the sequences in the SwissProt database. This comparison was performed using BLAST (version 2.13.0+) on a Linux machine with 36 cores due to the extensive data volume and processing time required (16 weeks for Nf wild‐type isoforms) (Camacho et al. 2009).

**FIGURE 2:**
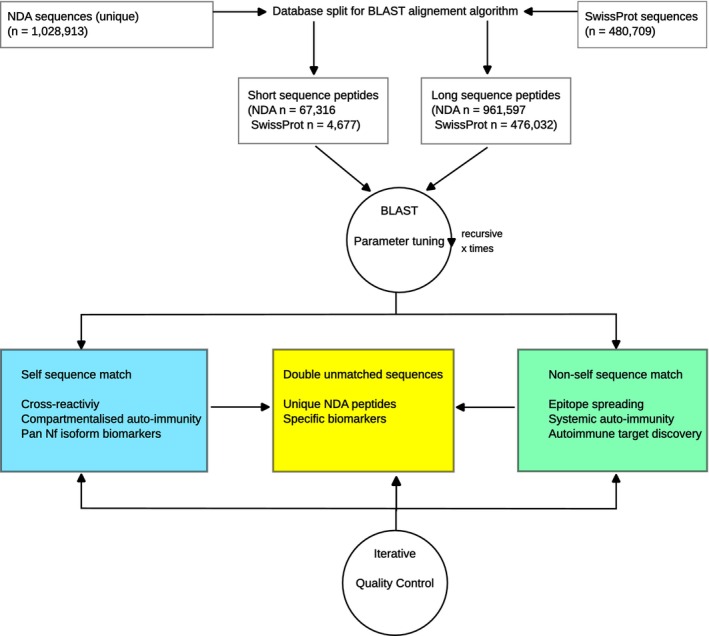
Peptide sequence alignment methodology. The neurofilament (Nf) isoform degradome atlas from Figure 4C and the SwissProtein database were divided according to peptide length: Short sequences (≤ 30 amino acids) and long sequences (> 30 amino acids). BLAST parameters were then optimised based on peptide length. A series of recursive BLAST analyses followed, with each iteration involving algorithm parameter adjustments, plus statistical and manual quality control. Three databases were generated for further analysis. The first database (blue) contained self‐matches within the Nf Degradome Atlas (NDA), enabling the discovery of pan‐Nf isoform biomarkers and the investigation of cross‐reactivity relevant to autoimmunity. The second database (green) included BLAST alignments between the NDA and the SwissProtein database, generating a list of candidate sequences for exploring autoimmunity and epitope spreading. The third database (yellow) comprised sequences uniquely associated with a particular Nf isoform. A notable limitation of this approach is that the number of enzymes capable of cleaving Nf is greater than those available in PeptideCutter. As discussed in the limitations section, this includes, among others, calpain (Nixon et al. 1990).

For short sequence alignments, the BLAST parameters were optimised using *‐task blastp‐short*, which included the scoring matrix PAM30. In the case of larger sequences, the scoring matrix BLOSUM62 was employed. Specifically, the parameter settings for short sequences entailed a cost of 9 for an open gap (1 for long sequences), a cost of 1 to extend a gap (also 1 for long sequences), a threshold of 16 (11), and a multiple hit window size of 11 (40). The query (word) length for recursive BLAST alignments initiated at 7 and gradually decreased in a stepwise manner with each recursive BLAST alignment, ultimately reaching a query length of 3 (Figure 2).

The *E*‐value was maintained at its default value of 10 to achieve maximum coverage for self‐sequence matches. After each recursive BLAST alignment, the hits were merged into the corresponding database for quality control. Only those sequences without any hits were included in the subsequent recursive step of BLAST alignment, employing the aforementioned stepwise reduction of query length from 7 to 3 (Figure 2). Consequently, the bitscore was utilized for comparing the hits instead of the E‐value, as the bitscore remains unaffected by varying database sizes. With each recursive BLAST alignment step, the database size was reduced by filtering out non‐matching sequences.

### 2.12 Peptide Structures

The NDA peptide structures were modelled using AlphaFold2 on ColabFold (release version 2022/4/29) (Mirdita et al. 2021). Only the highest‐ranked models generated by the AlphaFold2 algorithm were post‐processed. Post‐processing was performed in PyMol (version 2.5.0) with the incorporation of posttranslational modifications (PTMs) using PyTMs (Schrödinger LLC 2015; Warnecke et al. 2014). Amyloidogenic properties (APR) of individual NDA peptide sequences were tested using a validated, freely available high‐throughput algorithm called Aggregation Nucleation Prediction in Peptides and Proteins (ANuPP) (Prabakaran et al. 2021). The default threshold of 0.52 was kept.

### 2.13 Quality Control Pipeline

Quality control was performed manually and statistically in SAS. First, BLAST alignment and database mergers were done twice for the query database: (a) individually for each Nf isoform and (b) for Nf isoforms concatenated. Second, each database was revised manually for integrity. Third, each database was subjected to sequence comparison and duplicate elimination, with a final check on numbers adding up between manual and statistical quality control (Figure 2).

### 2.14 Datasets for Validating the NDA

For validation of the *m*/*z* peaks of the NDA, separate mass spectroscopy datasets were downloaded from the publicly available PRIDE repository: PXD039808, PXD014178, PRD000018 and PXD039414.

## 3 Results

### 3.1 Proteolysis

**FIGURE 3:**
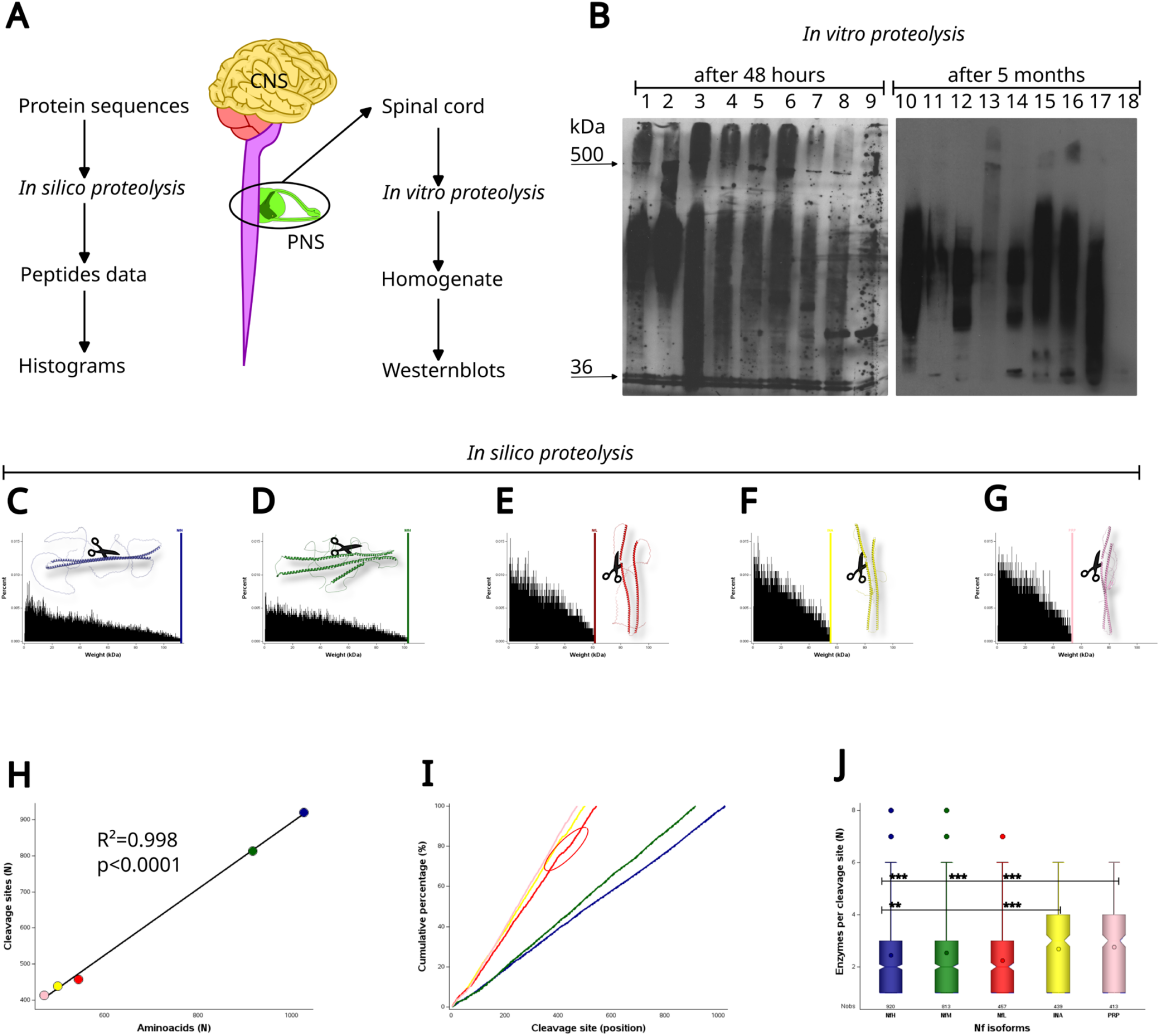
Complete proteolysis of neurofilament isoforms. (A) Overview of the experiments for comparison of proteolysis performed in vitro with proteolysis calculated in silico. (B) In vitro proteolysis of human spinal cord tissue. The Western blots show the extensive auto‐proteolysis of spinal cord tissue occurring within 48 h (Lanes 1–9). Prolonged proteolysis for 5 months reveals more extensive breakdown of peptides in the higher molecular range. Lane 1 (SMI35), 2 (SMI310), 3 (NR4), 4 (NF‐09), 5 (SMI34), 6 (SMI32), 7 (2E3), 8 (P5117), 9 (SMI26), 10 (SMI35), 11 (SMI38), 12 (SMI34), 13 (SMI32), 14 (NF‐09), 15 (SMI310), 16 (2E3), 17 (P5117), and 18 (HRP labelled rabbit anti‐mouse). (C) For in silico proteolysis, the FASTA sequences of Nf isoforms (colour‐coded protein structure as insets) were cut (scissor symbol) at reported protease cleavage sites. For the resulting peptide sequences the molecular weight was calculated. The histogram for NfH shows the size of computed peptides from the in silico proteolysis (*x*‐axis) against their relative percentage contribution (*y*‐axis). The blue‐coloured vertical reference line shows the total molecular weight of NfH. (D) NfM (green), (E) NfL (red), (F) INA (yellow) and (G) PRP (pink). (H) There is a linear correlation (*R* ^2^ = 0.998, *p* < 0.0001) between the number of cleavage sites and the total Nf isoform protein length (colour coding of dots as for C–G). (I) The positions of the Nf isoform cleavage sites are evenly distributed. There is a linear increase for the 100% cumulative percentage with one exception. Around amino acid position 400 in NfL (circled area) the cumulative frequency curve transiently flattens, which indicates that there are fewer proteolytic cleavage sites. (J) PRP and INA have significantly more cleavage sites which are simultaneous targets to multiple proteolytic enzymes compared to NfH, NfM and NfL. The total number of observations (Nobs), median (notch), mean (open circle), 25%–75% CI (box), 1%–99% CI (whisker), and outliers (dots) are shown.

To give a visual impression and also perform a systematic and complete proteolysis of all Nf isoforms, two methods were combined: in vitro and in silico (Figure 3A). For the in vitro proteolysis, spinal cord tissue was chosen as the only site where all five Nf isoforms are expressed at a sufficient level for immunoblot analysis. The Western blot analyses show a broad molecular weight range of peptides following proteolysis. After 48 h, there are still Nf protein aggregates at the top of the gel (Figure 3B, all lanes) with cleavage products producing a smear (Figure 3B, lanes 1–9). After 5 months of proteolysis, the higher molecular weight aggregates disappeared (Figure 3B, lanes 10–17) with non‐phosphorylated isoforms more readily cleaved than phosphorylated isoforms, as previously described (Goldstein et al. 1987). Next, systematic in silico proteolysis was performed (Figure 3; Table 1). Enzymatic and chemical cleavage sites in the Nf isoforms (Table 2) were predicted by PeptideCutter (for the list of enzymes and chemicals, see online methods) (SIB Swiss Institute of Bioinformatics 2021). Cleavage sites that are simultaneous substrates to six or more enzymes were highlighted in colour in the amino acid sequence (Table 1). As a visual analogue to the immunoblots, all Nf isoform cleavage products were presented as histograms (Figure 3C–G). Consistent with the Western blot results, the number of cleavage products is too large to permit a human researcher to identify, visually, single Nf peptides and is strongly correlated with protein lengths (*R* ^2^ = 0.998, *p* < 0.0001, Figure 3H) with equal distribution over the length of each Nf isoform (Figure 3I). INA and PRP stand out as targets for multiple enzyme cleavage sites (Figure 3J). In summary, the diverse range of Nf isoform cleavage products from both in vitro and in silico datasets highlights the challenge of visually distinguishing individual peptides. Therefore, a novel approach for cleavage product identification is presented: the neurofilament degradome atlas (NDA).

**TABLE 2:** Positions of Nf isoform‐specific cleavage sites within individual protein sequences.

#	NfL	NfM	NfH	INA	PRP
8	—	291	292	—	—
7	279	375	376	—	—
6	71, 280, 382	5, 40, 75, 112, 121, 150, 173, 189, 220, 233, 292, 394, 415, 834, 890	77, 108, 185, 205, 229, 297, 367, 395	8, 75, 105, 108, 211, 221, 226, 286, 291, 389, 425, 470	78, 90, 108, 111, 117, 227, 286, 347, 389, 462
5	4, 14, 68, 83, 101, 104, 135, 139, 167, 178, 209, 222, 223, 258, 265, 284, 329, 336, 354, 368, 402, 419, 432	24, 35, 52, 60, 61, 67, 77, 83, 94, 95, 115, 156, 161, 178, 196, 215, 245, 246, 262, 270, 277, 296, 298, 330, 331, 341, 366, 369, 387, 403, 412, 446, 451, 463, 480, 601, 757, 798, 872, 893, 894	4, 10, 26, 28, 47, 50, 65, 111, 144, 146, 169, 174, 176, 240, 241, 260, 263, 278, 293, 331, 332, 335, 342, 349, 350, 370, 388, 404, 811, 904, 924, 983, 1019	3, 38, 64, 87, 125, 126, 141, 142, 143, 146, 148, 154, 165, 166, 168, 171, 201, 208, 213, 220, 223, 224, 237, 238, 239, 272, 275, 287, 336, 341, 342, 343, 361, 382, 398, 407, 446, 474	12, 32, 40, 45, 60, 70, 80, 89, 91, 103, 143, 147, 150, 156, 157, 162, 169, 172, 174, 176, 179, 183, 191, 199, 200, 212, 214, 221, 226, 228, 230, 237, 266, 272, 287, 291, 336, 342, 349, 361, 381, 382, 398, 439
4	6, 10, 33, 40, 43, 51, 73, 94, 97, 110, 121, 122, 123, 125, 144, 148, 151, 160, 171, 183, 186, 192, 204, 216, 217, 233, 234, 240, 247, 254, 268, 287, 335, 337, 357, 358, 375, 376, 389, 400, 412, 424, 427, 433, 442, 443, 481	3, 4, 8, 14, 16, 26, 63, 68, 84, 96, 101, 105, 108, 111, 117, 135, 136, 146, 155, 158, 167, 177, 179, 180, 184, 188, 190, 194, 195, 204, 205, 207, 208, 211, 227, 232, 234, 239, 258, 259, 261, 269, 289, 299, 315, 319, 320, 349, 359, 370, 380, 382, 383, 388, 401, 402, 408, 411, 414, 424, 452, 454, 458, 465, 470, 472, 473, 478, 479, 486, 493, 496, 502, 522, 526, 528, 534, 539, 549, 554, 560, 562, 567, 578, 584, 589, 593, 599, 608, 611, 623, 636, 649, 662, 675, 688, 703, 707, 715, 719, 722, 726, 730, 740, 758, 763, 767, 771, 773, 775, 784, 796, 810, 818, 823, 824, 841, 849, 859, 884, 887, 889, 891, 901	9, 11, 24, 30, 64, 79, 80, 97, 101, 103, 104, 107, 131, 132, 145, 148, 151, 157, 158, 159, 160, 167, 168, 173, 184, 192, 193, 200, 201, 204, 213, 216, 222, 223, 225, 230, 236, 242, 270, 271, 272, 275, 286, 287, 290, 294, 296, 299, 300, 316, 320, 321, 334, 339, 360, 371, 382, 383, 384, 389, 402, 403, 409, 412, 441, 448, 451, 458, 462, 468, 472, 476, 480, 485, 490, 498, 507, 513, 522, 534, 536, 548, 556, 568, 570, 582, 590, 602, 610, 624, 636, 644, 650, 656, 662, 664, 670, 684, 698, 706, 712, 720, 726, 734, 740, 748, 754, 760, 764, 765, 778, 789, 797, 803, 817, 824, 832, 849, 860, 863, 868, 881, 889, 901, 913, 930, 952, 959, 961, 965, 972, 987, 998, 1001, 1009, 1015	15, 16, 24, 25, 28, 29, 43, 60, 62, 66, 77, 86, 94, 98, 101, 104, 107, 110, 120, 121, 128, 129, 138, 145, 151, 155, 164, 173, 177, 178, 179, 181, 183, 189, 194, 195, 207, 210, 214, 225, 233, 241, 242, 261, 264, 265, 266, 269, 284, 294, 306, 309, 310, 314, 329, 344, 354, 365, 375, 377, 378, 383, 396, 397, 403, 406, 412, 430, 431, 444, 449, 471, 473, 475, 485, 490	8, 9, 17, 31, 34, 39, 44, 56, 57, 64, 69, 71, 77, 85, 86, 97, 101, 104, 107, 113, 116, 124, 125, 129, 139, 148, 149, 153, 158, 160, 164, 178, 182, 184, 188, 190, 198, 201, 202, 208, 209, 211, 216, 222, 233, 235, 236, 261, 264, 265, 269, 284, 294, 305, 322, 329, 335, 337, 341, 344, 348, 354, 360, 362, 364, 365, 370, 375, 377, 378, 383, 396, 397, 403, 406, 414, 417, 435
3	9, 18, 57, 72, 82, 90, 96, 111, 117, 118, 124, 137, 145, 156, 164, 168, 170, 179, 180, 182, 185, 191, 193, 196, 199, 203, 210, 213, 228, 229, 252, 257, 259, 266, 267, 277, 278, 286, 295, 302, 312, 316, 317, 328, 330, 342, 353, 369, 371, 372, 374, 390, 391, 393, 396, 418, 449, 455, 464, 467, 477, 478, 479, 480, 484, 487, 488, 489, 493, 494, 495, 500, 503, 507, 508, 509, 528, 542	7, 15, 19, 21, 34, 36, 42, 54, 56, 72, 74, 76, 82, 98, 102, 103, 107, 118, 122, 123, 127, 128, 130, 132, 137, 153, 162, 163, 168, 172, 185, 186, 187, 191, 197, 200, 214, 217, 219, 222, 223, 226, 230, 235, 240, 243, 244, 257, 264, 271, 272, 274, 278, 290, 293, 295, 301, 304, 307, 308, 311, 314, 321, 322, 332, 335, 338, 339, 342, 343, 344, 351, 355, 365, 367, 371, 374, 376, 377, 379, 384, 386, 391, 392, 397, 404, 405, 406, 422, 435, 440, 442, 445, 447, 461, 466, 468, 469, 484, 485, 487, 488, 489, 490, 494, 495, 497, 500, 503, 504, 509, 514, 519, 521, 523, 527, 529, 530, 535, 536, 537, 538, 541, 555, 556, 561, 565, 570, 572, 574, 576, 581, 583, 585, 586, 587, 590, 598, 603, 606, 612, 624, 637, 650, 663, 676, 677, 693, 695, 700, 702, 704, 705, 706, 708, 710, 711, 714, 716, 717, 720, 721, 723, 731, 732, 733, 735, 739, 745, 754, 759, 760, 762, 772, 774, 776, 780, 785, 797, 805, 807, 809, 813, 815, 817, 825, 826, 827, 833, 835, 839, 842, 850, 851, 860, 864, 871, 882, 892, 898, 899, 902, 910, 911	1, 3, 7, 14, 15, 17, 23, 38, 46, 52, 66, 74, 76, 85, 88, 95, 98, 99, 112, 114, 116, 117, 118, 119, 123, 124, 125, 126, 128, 133, 139, 147, 149, 152, 154, 164, 170, 172, 175, 182, 186, 188, 190, 196, 202, 208, 210, 214, 218, 219, 226, 231, 258, 264, 273, 279, 291, 302, 305, 308, 312, 322, 323, 328, 333, 343, 344, 346, 348, 352, 355, 362, 366, 372, 375, 377, 378, 381, 385, 387, 392, 393, 398, 405, 406, 407, 410, 415, 419, 420, 422, 424, 426, 437, 442, 443, 445, 452, 453, 473, 474, 475, 477, 479, 481, 484, 486, 491, 492, 495, 499, 514, 515, 521, 529, 535, 543, 549, 555, 563, 569, 577, 583, 589, 597, 603, 609, 617, 623, 631, 637, 643, 651, 657, 663, 671, 677, 679, 685, 691, 693, 699, 705, 713, 719, 727, 733, 741, 747, 755, 761, 771, 777, 781, 784, 787, 790, 796, 804, 809, 810, 818, 819, 822, 823, 831, 833, 834, 838, 840, 842, 843, 846, 847, 850, 851, 852, 861, 862, 866, 867, 872, 873, 874, 879, 882, 883, 884, 885, 892, 893, 895, 897, 899, 900, 906, 907, 914, 915, 919, 921, 923, 929, 933, 936, 937, 938, 940, 943, 945, 946, 953, 954, 955, 960, 964, 966, 967, 969, 973, 978, 980, 982, 984, 986, 990, 991, 996, 999, 1006, 1016, 1021	2, 6, 9, 17, 19, 37, 39, 42, 45, 59, 61, 63, 67, 74, 76, 83, 88, 91, 95, 96, 100, 111, 114, 115, 116, 122, 123, 130, 132, 135, 149, 161, 167, 184, 187, 190, 193, 196, 197, 203, 215, 216, 218, 219, 228, 251, 252, 259, 267, 273, 274, 279, 285, 290, 293, 296, 300, 303, 315, 316, 317, 322, 325, 327, 328, 330, 334, 335, 337, 338, 346, 350, 360, 362, 364, 366, 371, 372, 374, 379, 381, 386, 387, 392, 399, 400, 401, 411, 416, 438, 443, 445, 447, 448, 450, 451, 452, 453, 457, 461, 468, 469, 482, 498	7, 18, 19, 21, 59, 61, 72, 73, 76, 82, 84, 94, 100, 110, 114, 118, 119, 126, 128, 133, 136, 140, 142, 152, 154, 155, 161, 168, 175, 185, 194, 196, 197, 204, 205, 213, 215, 217, 219, 220, 224, 229, 234, 238, 244, 252, 254, 260, 273, 279, 282, 285, 292, 293, 300, 303, 306, 311, 314, 316, 317, 321, 325, 326, 328, 330, 334, 338, 339, 343, 346, 350, 363, 366, 367, 368, 369, 372, 374, 379, 386, 387, 392, 399, 400, 401, 413, 416, 425, 427, 444, 446, 449, 453, 456, 457, 459, 461, 470
2	1, 3, 7, 16, 17, 19, 20, 23, 30, 37, 50, 54, 55, 63, 64, 69, 70, 81, 87, 95, 98, 100, 103, 105, 106, 107, 109, 112, 119, 120, 126, 131, 134, 136, 138, 140, 142, 146, 147, 150, 163, 165, 169, 172, 173, 174, 177, 181, 188, 189, 194, 200, 201, 206, 207, 208, 212, 215, 218, 219, 221, 224, 227, 230, 232, 245, 246, 250, 262, 270, 273, 276, 283, 288, 289, 292, 299, 301, 303, 304, 307, 309, 310, 311, 314, 318, 319, 320, 321, 323, 325, 327, 332, 333, 338, 347, 348, 355, 359, 361, 364, 365, 367, 370, 373, 378, 380, 384, 385, 388, 392, 394, 397, 399, 421, 426, 434, 437, 450, 452, 453, 454, 456, 458, 459, 461, 463, 465, 466, 468, 469, 473, 475, 476, 482, 483, 485, 486, 490, 492, 496, 498, 499, 501, 504, 505, 506, 510, 515, 518, 521, 524, 525, 526, 527, 535, 540, 541	1, 13, 18, 47, 53, 57, 66, 71, 92, 93, 109, 116, 129, 131, 139, 143, 147, 149, 157, 160, 166, 175, 192, 199, 201, 202, 206, 210, 212, 216, 218, 221, 228, 229, 231, 238, 241, 242, 253, 255, 256, 263, 265, 268, 281, 285, 288, 297, 300, 302, 303, 309, 313, 316, 317, 318, 324, 327, 329, 337, 345, 346, 348, 350, 354, 356, 360, 362, 373, 381, 385, 390, 393, 396, 398, 399, 400, 409, 423, 427, 431, 441, 450, 453, 455, 456, 457, 459, 460, 462, 464, 471, 474, 475, 477, 481, 491, 492, 498, 501, 505, 506, 507, 508, 510, 513, 520, 524, 531, 540, 543, 544, 545, 548, 550, 563, 568, 571, 573, 575, 579, 580, 582, 588, 591, 594, 595, 597, 600, 602, 604, 605, 607, 610, 613, 614, 619, 622, 625, 627, 632, 635, 638, 640, 645, 648, 651, 653, 658, 661, 664, 666, 671, 674, 678, 679, 684, 687, 689, 690, 691, 694, 698, 709, 718, 725, 734, 738, 741, 742, 743, 744, 746, 748, 750, 751, 753, 756, 761, 764, 768, 786, 788, 789, 790, 792, 793, 800, 801, 806, 811, 812, 816, 819, 829, 836, 840, 843, 845, 846, 847, 852, 853, 855, 856, 857, 858, 866, 870, 874, 875, 878, 883, 885, 886, 888, 897, 900, 906, 907, 908, 909, 915	2, 18, 25, 27, 31, 40, 70, 89, 90, 91, 105, 113, 129, 130, 141, 142, 153, 156, 162, 178, 180, 187, 194, 195, 197, 198, 209, 211, 232, 237, 250, 255, 257, 262, 265, 281, 282, 303, 304, 310, 311, 317, 319, 325, 330, 336, 338, 357, 363, 369, 374, 379, 386, 391, 394, 397, 399, 400, 401, 428, 435, 436, 438, 439, 444, 446, 447, 449, 454, 455, 456, 457, 459, 461, 463, 467, 469, 471, 478, 482, 487, 493, 494, 500, 501, 502, 506, 508, 509, 516, 517, 520, 523, 524, 525, 528, 530, 531, 537, 538, 539, 542, 544, 545, 550, 551, 554, 557, 558, 559, 564, 565, 571, 572, 573, 576, 578, 579, 584, 585, 588, 591, 592, 593, 596, 598, 599, 604, 605, 608, 611, 612, 613, 616, 618, 619, 622, 625, 626, 627, 630, 632, 633, 638, 639, 642, 645, 646, 647, 652, 653, 658, 659, 665, 666, 667, 672, 673, 676, 678, 680, 681, 686, 687, 690, 692, 694, 695, 700, 701, 704, 707, 708, 709, 714, 715, 718, 721, 722, 723, 728, 729, 732, 735, 736, 737, 742, 743, 746, 749, 750, 751, 756, 757, 762, 763, 767, 768, 772, 773, 776, 779, 785, 786, 791, 792, 795, 798, 799, 800, 805, 806, 813, 814, 825, 826, 827, 830, 835, 839, 841, 848, 858, 859, 869, 878, 880, 887, 888, 891, 896, 898, 902, 903, 905, 908, 918, 920, 922, 926, 928, 931, 932, 934, 935, 939, 942, 944, 950, 951, 963, 968, 971, 975, 976, 977, 979, 981, 985, 988, 995, 997, 1000, 1002, 1018, 1020, 1022, 1023, 1024, 1026	1, 7, 18, 20, 48, 52, 53, 65, 68, 72, 80, 81, 89, 102, 106, 109, 124, 127, 133, 134, 147, 150, 156, 172, 174, 185, 192, 217, 222, 231, 232, 234, 235, 236, 248, 249, 250, 255, 256, 257, 276, 277, 278, 280, 283, 288, 298, 302, 304, 311, 313, 319, 323, 324, 326, 340, 345, 349, 351, 355, 363, 368, 369, 376, 380, 385, 388, 391, 393, 394, 395, 404, 417, 426, 432, 454, 458, 462, 472, 476, 477, 478, 481, 483, 484, 486, 489, 491, 493	1, 20, 27, 46, 83, 98, 105, 112, 123, 151, 167, 173, 177, 180, 186, 189, 193, 218, 223, 225, 231, 232, 239, 243, 251, 253, 256, 257, 259, 262, 263, 275, 276, 277, 278, 280, 283, 288, 290, 295, 297, 302, 304, 308, 309, 312, 323, 340, 345, 358, 371, 376, 385, 388, 391, 393, 395, 404, 419, 420, 426, 430, 436, 437, 438, 440, 441, 442, 443, 450, 452, 463, 464
1	5, 8, 12, 13, 15, 21, 24, 25, 26, 29, 35, 36, 39, 42, 45, 47, 53, 65, 76, 77, 78, 79, 84, 86, 88, 89, 91, 92, 99, 102, 108, 115, 116, 127, 128, 129, 130, 141, 143, 149, 152, 153, 154, 155, 157, 159, 162, 176, 187, 195, 197, 202, 205, 211, 214, 220, 225, 226, 231, 236, 238, 239, 241, 243, 248, 249, 251, 253, 256, 260, 261, 263, 269, 271, 281, 285, 291, 293, 296, 297, 298, 300, 305, 306, 313, 315, 326, 331, 334, 339, 343, 344, 346, 349, 350, 351, 352, 356, 360, 362, 363, 366, 379, 381, 383, 386, 387, 395, 398, 403, 405, 408, 409, 411, 423, 429, 431, 436, 439, 444, 446, 447, 448, 457, 460, 462, 472, 474, 491, 497, 502, 512, 513, 514, 516, 517, 519, 520, 522, 523, 529, 530, 531, 534, 536, 539, 543	2, 6, 12, 17, 20, 23, 27, 39, 48, 51, 55, 58, 62, 65, 70, 85, 100, 104, 110, 113, 114, 119, 120, 126, 133, 134, 138, 140, 141, 144, 145, 148, 151, 152, 154, 159, 164, 169, 170, 171, 174, 176, 181, 182, 183, 193, 198, 203, 209, 213, 224, 247, 248, 249, 250, 251, 254, 260, 266, 273, 276, 280, 282, 284, 286, 287, 306, 310, 312, 323, 326, 328, 333, 334, 340, 347, 352, 358, 361, 363, 368, 378, 389, 395, 407, 410, 416, 418, 419, 420, 425, 426, 429, 430, 432, 434, 436, 437, 438, 439, 443, 444, 448, 467, 476, 482, 483, 499, 512, 515, 516, 517, 518, 525, 533, 542, 546, 547, 553, 559, 564, 566, 569, 577, 592, 596, 609, 617, 618, 621, 626, 630, 631, 634, 639, 643, 644, 647, 652, 656, 657, 660, 665, 668, 669, 670, 673, 682, 683, 686, 692, 696, 697, 699, 701, 712, 713, 724, 727, 728, 729, 737, 747, 749, 752, 755, 765, 766, 770, 777, 779, 783, 791, 799, 802, 803, 804, 808, 814, 828, 830, 831, 832, 838, 848, 854, 861, 862, 863, 867, 868, 869, 873, 876, 877, 879, 880, 881, 895, 903, 904, 912, 913, 916	6, 8, 13, 16, 19, 29, 33, 34, 37, 39, 41, 48, 49, 51, 53, 54, 55, 57, 58, 59, 60, 67, 68, 69, 71, 75, 78, 82, 84, 86, 87, 92, 93, 96, 100, 102, 106, 109, 110, 115, 120, 121, 127, 136, 137, 140, 143, 150, 155, 161, 163, 166, 171, 177, 179, 181, 189, 199, 203, 206, 207, 212, 215, 217, 220, 221, 224, 228, 233, 234, 235, 238, 239, 244, 245, 249, 251, 252, 253, 254, 256, 259, 261, 266, 267, 268, 269, 274, 276, 277, 283, 289, 295, 298, 301, 306, 309, 313, 314, 315, 318, 324, 326, 327, 329, 340, 341, 347, 351, 353, 354, 356, 358, 359, 361, 364, 368, 380, 390, 396, 408, 413, 414, 418, 423, 427, 429, 431, 432, 434, 440, 450, 464, 465, 466, 470, 483, 489, 497, 505, 510, 512, 519, 527, 533, 541, 547, 553, 562, 567, 575, 581, 587, 595, 601, 607, 615, 621, 629, 635, 649, 655, 661, 669, 675, 683, 689, 697, 703, 711, 717, 725, 731, 738, 739, 745, 753, 759, 766, 770, 774, 775, 780, 783, 788, 794, 802, 808, 812, 815, 816, 820, 821, 829, 836, 837, 845, 853, 854, 855, 856, 857, 864, 865, 870, 871, 875, 876, 877, 886, 890, 894, 909, 911, 912, 916, 917, 925, 927, 941, 948, 949, 956, 957, 958, 962, 970, 974, 989, 993, 994, 1007, 1008, 1010, 1011, 1012, 1014, 1017, 1025	5, 14, 21, 26, 27, 31, 32, 34, 35, 47, 49, 51, 56, 57, 70, 71, 73, 79, 82, 84, 85, 90, 92, 93, 97, 103, 112, 113, 117, 139, 140, 152, 153, 157, 159, 160, 163, 169, 170, 175, 180, 182, 191, 198, 202, 204, 205, 206, 209, 212, 227, 229, 230, 240, 243, 244, 247, 253, 254, 258, 260, 262, 263, 268, 270, 282, 289, 292, 295, 297, 299, 305, 307, 312, 318, 320, 321, 331, 332, 333, 339, 347, 348, 352, 353, 356, 357, 358, 370, 373, 384, 390, 402, 405, 409, 413, 414, 420, 427, 433, 434, 435, 436, 437, 439, 442, 455, 456, 459, 460, 463, 465, 466, 467, 479, 488, 492, 494, 497, 499	3, 5, 10, 11, 15, 16, 26, 30, 33, 48, 49, 54, 55, 65, 66, 67, 68, 75, 79, 81, 88, 92, 93, 96, 99, 102, 106, 109, 115, 122, 127, 131, 132, 135, 137, 138, 141, 146, 163, 165, 166, 170, 171, 187, 192, 195, 203, 206, 207, 240, 241, 242, 247, 248, 250, 255, 258, 267, 268, 270, 274, 281, 289, 296, 298, 307, 310, 313, 318, 319, 324, 327, 332, 333, 351, 353, 355, 356, 357, 359, 373, 380, 384, 390, 394, 402, 407, 409, 410, 411, 412, 415, 418, 421, 422, 423, 424, 431, 433, 445, 447, 448, 451, 455, 460, 466, 467, 468, 469

*Note:* The data in this table are arranged in descending order based on the number of enzymes (#) that potentially cleave each protein sequence position.

### 3.2 NDA

The first step in developing the NDA is to quantitatively organise the in silico Nf degradome. Mathematically, this is done through combinatorics. There are $Z=\left(\frac{\left(x+1\right)\times \left(x+2\right)}{2}-1\right)$ cleavage products, for *x* cleavage sites, in any given protein. Therefore, the number of current Nf isoform cleavage products in the NDA (NfH + NfM + NfL + INA + PRP, Table 3) calculates as:

$$\begin{array}{c} Z=\left(\frac{\left(920+1\right)\times \left(920+2\right)}{2}-1\right)\\ +\left(\frac{\left(813+1\right)\times \left(813+2\right)}{2}-1\right)\\ +\left(\frac{\left(457+1\right)\times \left(457+2\right)}{2}-1\right)\\ +\left(\frac{\left(439+1\right)\times \left(439+2\right)}{2}-1\right)\\ +\left(\frac{\left(413+1\right)\times \left(413+2\right)}{2}-1\right)\\ 1\;044\;317 \end{array}$$

**FIGURE 4:**
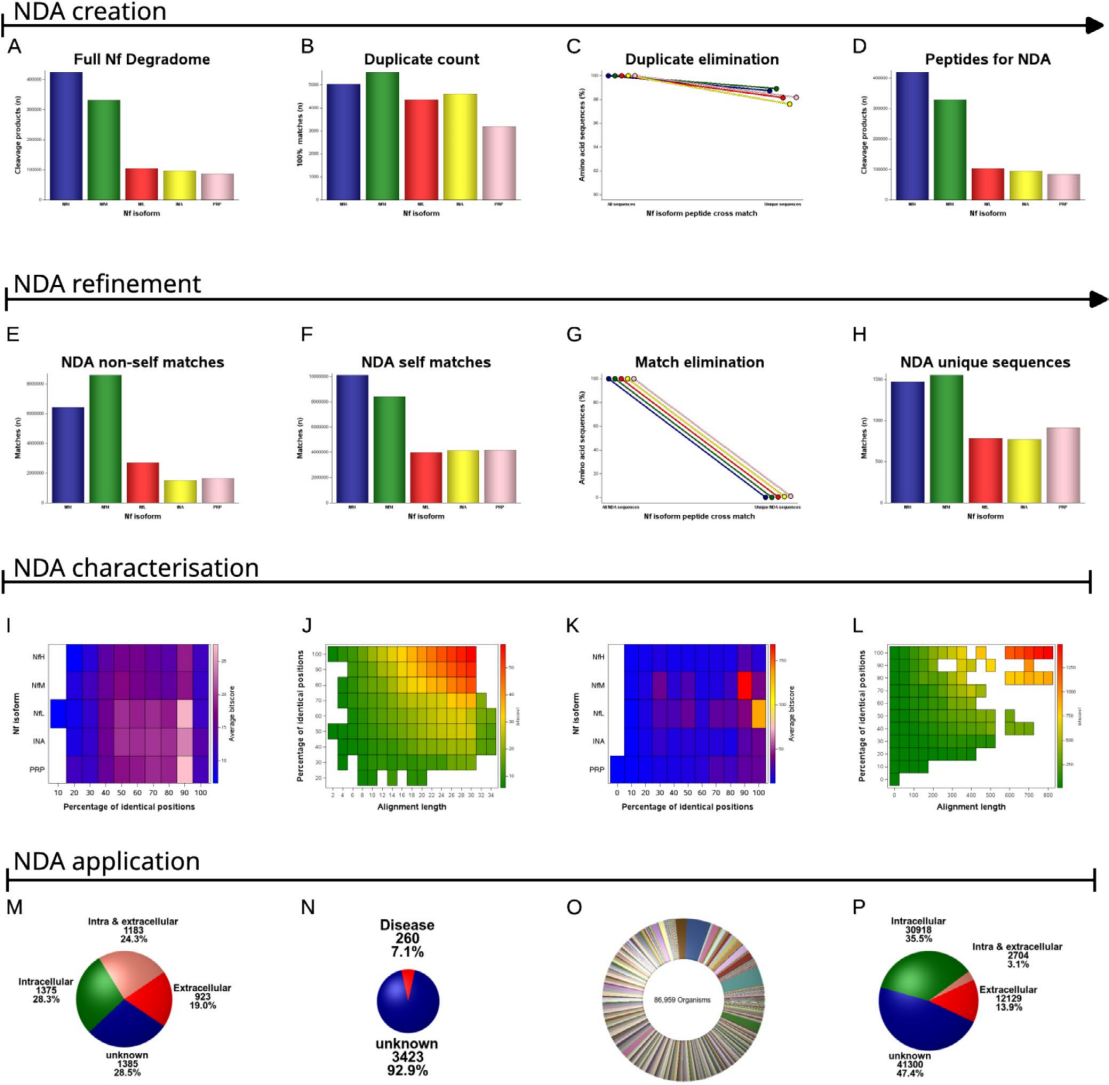
The neurofilament degradome atlas (NDA). (A) The human Nf degradome contains 1 044 317 peptides. (B) Duplicate counts between Nf isoforms are characterised by a 100% identical match of the amino acid sequence. These matches are useful for Nf biomarker assay development aimed at the highest sensitivity for detecting neurodegeneration. These peptides are defined as the pan‐Nf sequences. (C) Duplicate elimination reduces the Nf degradome by about 1%–3% per Nf isoform. (D) The NDA contains 1 029 453 Nf isoform peptides. This database is made available as a research resource and annotated as explained in sub figures (E)–(P). (E) There are 20 861 579 BLAST matches between the NDA and the human proteome. These matches identify peptides that could be the source of false positives in a biomarker assay due to non‐specific cross‐reactivity with other proteins. (F) There are 30 841 923 BLAST self‐matches between the Nf isoforms themselves. These matches identify the non‐Nf isoform‐specific NDA peptides. (G) Elimination of non‐self and self‐matches reduces the number of peptides in the NDA by 97.6%–98.9% per Nf isoform. (H) There are 5492 peptides in the NDA that are specific for single Nf isoforms. These unique NDA peptides are of interest for Nf isoform‐targeted biomarker assay development. (I) The heatmap represents, for each Nf isoform, the quality of the BLAST non‐self matches. The bitscore is shown because it is stable for comparison of differently sized databases. The highest‐quality non‐self matches are found for NfL and PRP. Note that the top 100% identical matches do not show in this subfigure because they were already eliminated in subfigure (C). (J) The BLAST alignment length indicates that the highest scoring matches, across all species, were found for proteins genetically related to the human Nf isoforms (highlighted in red in the top right corner). These NDA peptides may be considered for experimental models requiring NF isoforms as biomarkers for neurodegeneration. (K) For the NDA, the quality of the self‐matches is highest for NfM and NfL. The highest quality matches should be considered for investigation of epitope spreading in suspected Nf autoimmune disease. (L) The highest‐quality self‐matches are for highly conserved sequences of over 600 amino acids in length, which are common to all Nf isoforms (top right corner of the figure). These matches identify NDA peptides useful for pan‐Nf antibody development, expanding on the 100% identity matches shown in (B). (M) The human non‐self matches of the NDA were reviewed to identify the cellular location of the potential autoimmune target. For studying autoimmunity, most relevant are the 19%–24% extracellular targets. (N) Human diseases were associated with 7.1% of the non‐self matches. These NDA matches should be considered for the investigation of causality in Nf autoimmunity through epitope spreading following disease. (O) The NDA had matches with peptides from 86 959 non‐human organisms. These matches should be considered in biomarker experiments where contamination with non‐human material is possible. For example, paleoproteomics. (P) Among the non‐human matches, 17% of the peptides contained an extracellular component. These matches warrant consideration for investigating causality in Nf autoimmunity triggered through epitope spreading following contact.

**FIGURE 5:**
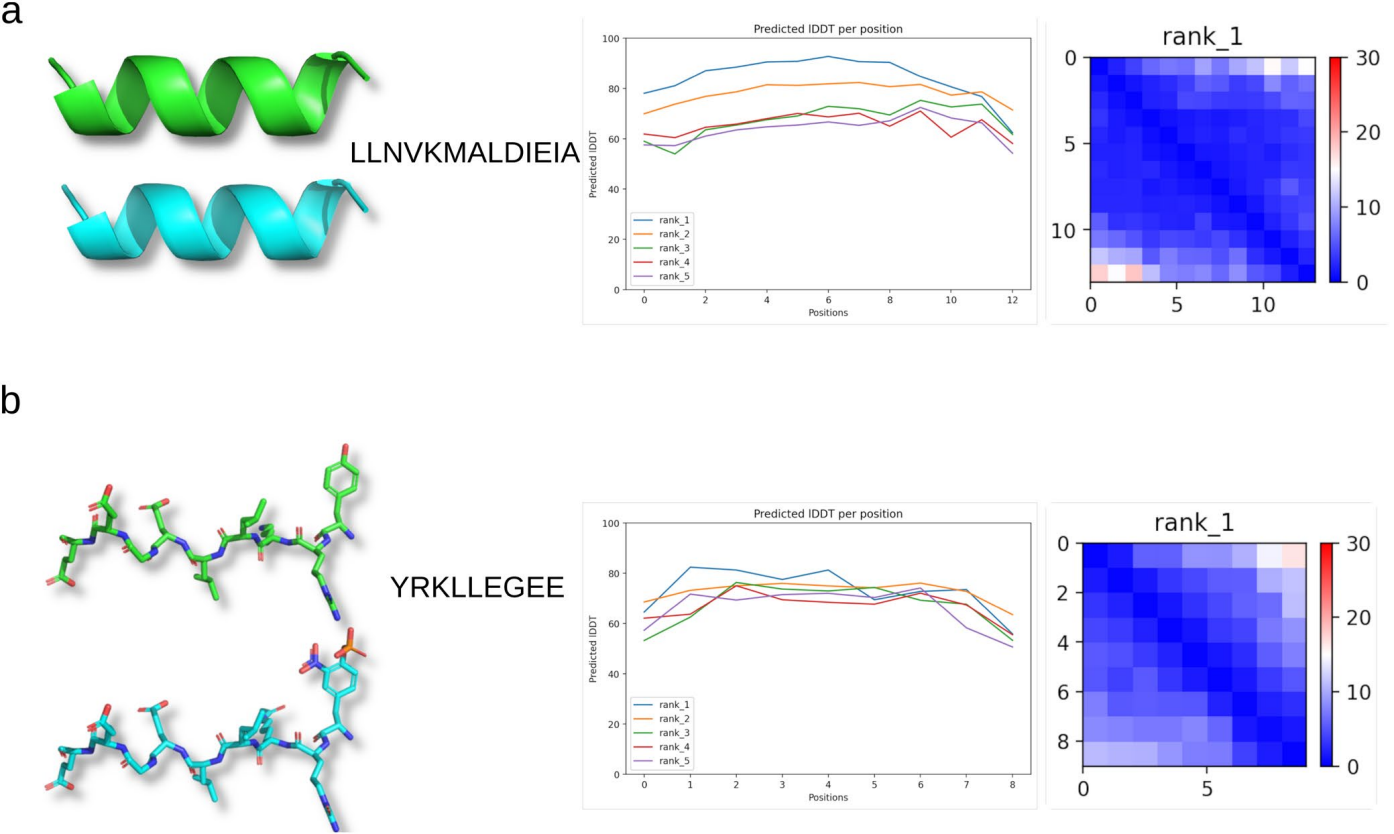
Pan‐Nf peptide structures. Table 4 was reviewed to identify the longest pan‐Nf isoform peptides. The results for protein structure modelling with AlphaFold2 are shown for the two largest sequences, which also recur in smaller fragments: (a) LLNVKMALDIEIA and (b) YRKLLEGEE. The line plots for reach model and the predicted local distance difference test (lDDT) per position are shown.

Biochemically, a proportion of these Nf isoform cleavage products is of identical amino acid sequence. These duplicates (Figure 4B) account for 1%–2% of all Nf amino acid sequences (Figure 4C) and are here defined as pan‐Nf sequences. Figure 5 shows two representative examples, with the largest pan‐Nf sequences being listed in Table 4. Next, duplicate pan‐Nf sequences were removed from the NDA (Figure 4D).

**TABLE 3:** The total count of Nf isoform‐specific cleavage sites, categorized by the number of enzymes potentially involved in cleaving at each position in the protein sequence.

Number of	Sum of cleavage sites				
enzymes	NfH	NfM	NfL	INA	PRP
8	1	1	—	—	—
7	1	1	1	—	—
6	8	15	3	12	10
5	33	41	23	38	44
4	136	124	47	76	78
3	232	204	78	104	99
2	270	223	158	89	73
1	239	204	147	120	109
Total	∑920	∑813	∑457	∑439	∑413

*Note:* For detailed information on the specific protein sequence positions where cleavage occurs, refer to Table 2. The total number of cleavage sites determines the size of the NDA.

**FIGURE 6:**
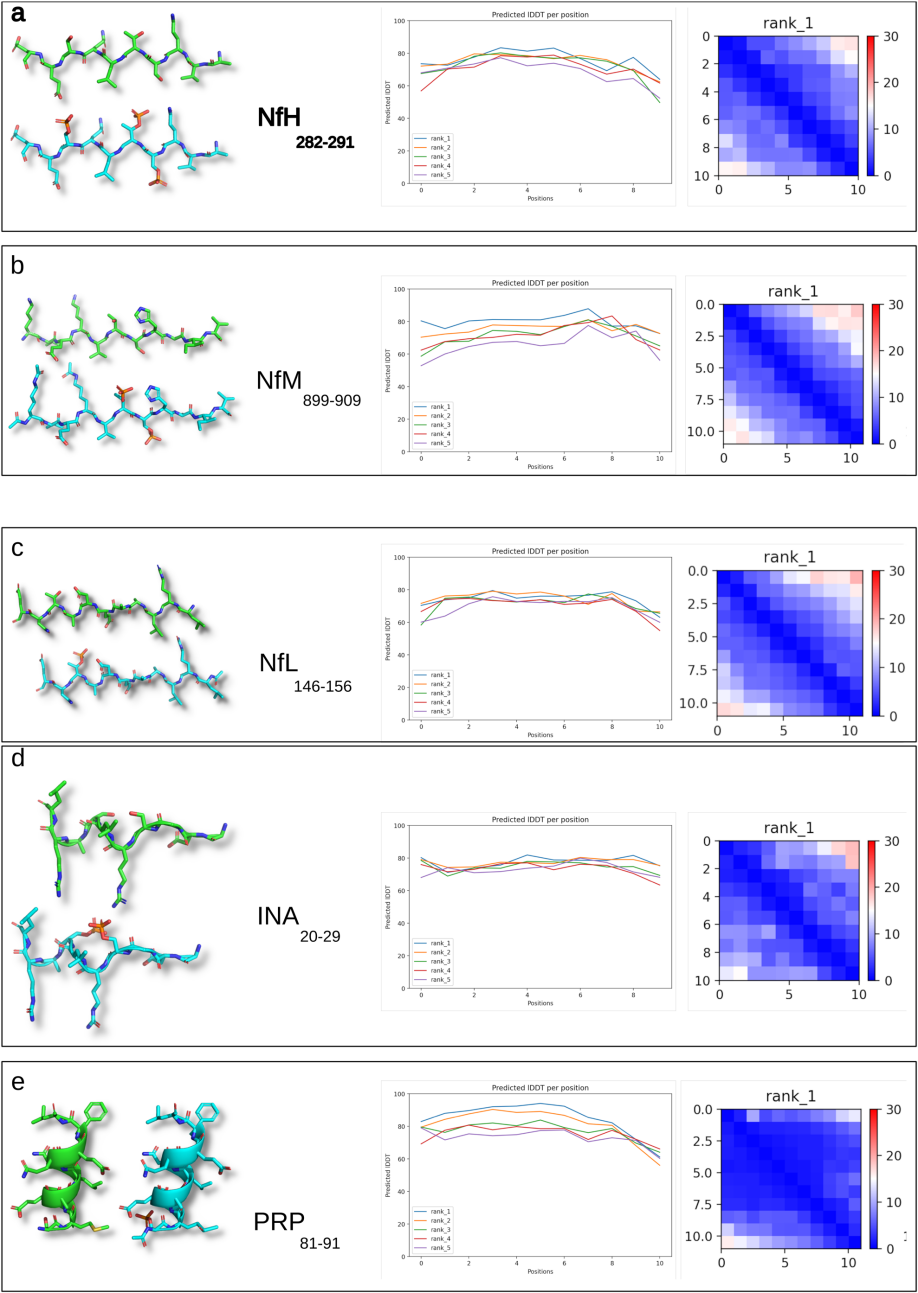
Examples of diverse structures from representative unique NDA peptides. The NDA was reviewed to identify, for each Nf isoform, the longest unique amino acid sequence. The results for protein structure modeling with AlphaFold2 are shown for (a) NfH282–291, (b) NfM899–909, (c) NfL146–156, (d) INA20–92, and (e) PRP81–91. The structure of both the naked (green coloured) and posttranslational modification‐altered (blue coloured) PyMol sculptured models is shown for each Nf isoform. The line plots for each model (a–e) show the predicted lDDT per position, which denotes the estimated accuracy of the predicted structures at each residue position. Higher lDDT values signify greater confidence in the predicted structure at that specific position.

In the third step of creating the NDA, I address analytical specificity and sensitivity, recognising that maximising one can diminish the other (Khalil et al. 2024; Shaw et al. 2023). I propose that the highest analytical specificity for an Nf biomarker assay arises from NDA peptides uniquely found in the human proteome (Figure 4E) and from removing peptides shared among Nf isoforms (Figure 4F). Optimisation of the NDA for specificity requires the elimination of 97% of peptides (Figure 4G). This leaves a small selection of completely unique Nf isoform amino acid sequences (Figure 4H). Figure 6 shows representative examples of unique sequences for each of the Nf isoforms.

The fourth step focuses on the associations of the NDA with disease. This involves reviewing the amino acid similarities between the NDA and the human proteome (Figure 4I), emphasising the need for careful testing for cross‐reactivity in biomarker test development. Practically, high‐quality matches were almost exclusively with other intermediate filament proteins (Figure 4J), a particular issue for NfM and NfL (Figure 4K). Narrowing the NDA selection further down, Figure 4L reveals an isolated cluster of high bitscore, large alignment self‐matches specific to preserved rod domains common to all Nf isoforms. In order to expand the NDA to identify experimental targets in autoimmunity, matches with diseases and pathogens are conducted (Figure 4M–P). There were 4868 non‐self matches identified with human proteins. Mapping of these matches with UniProt's ID mapping demonstrates that most were with at least one extracellular component (43.3%, Figure 4M); few were related to known human diseases (Figure 4N), with a large undisclosed portion of potential pathogens (Figure 4O) that harbour autoimmunity‐relevant extracellular epitopes (Figure 4P).

### 3.3 Validation of the Datasets

It was suggested that in silico cleavage can supplement in vitro proteolysis (Figure 3). The preceding section was dedicated to structuring the comprehensive data derived (Figure 4). However, it is imperative to validate the in silico data with in vitro data.

**FIGURE 7:**
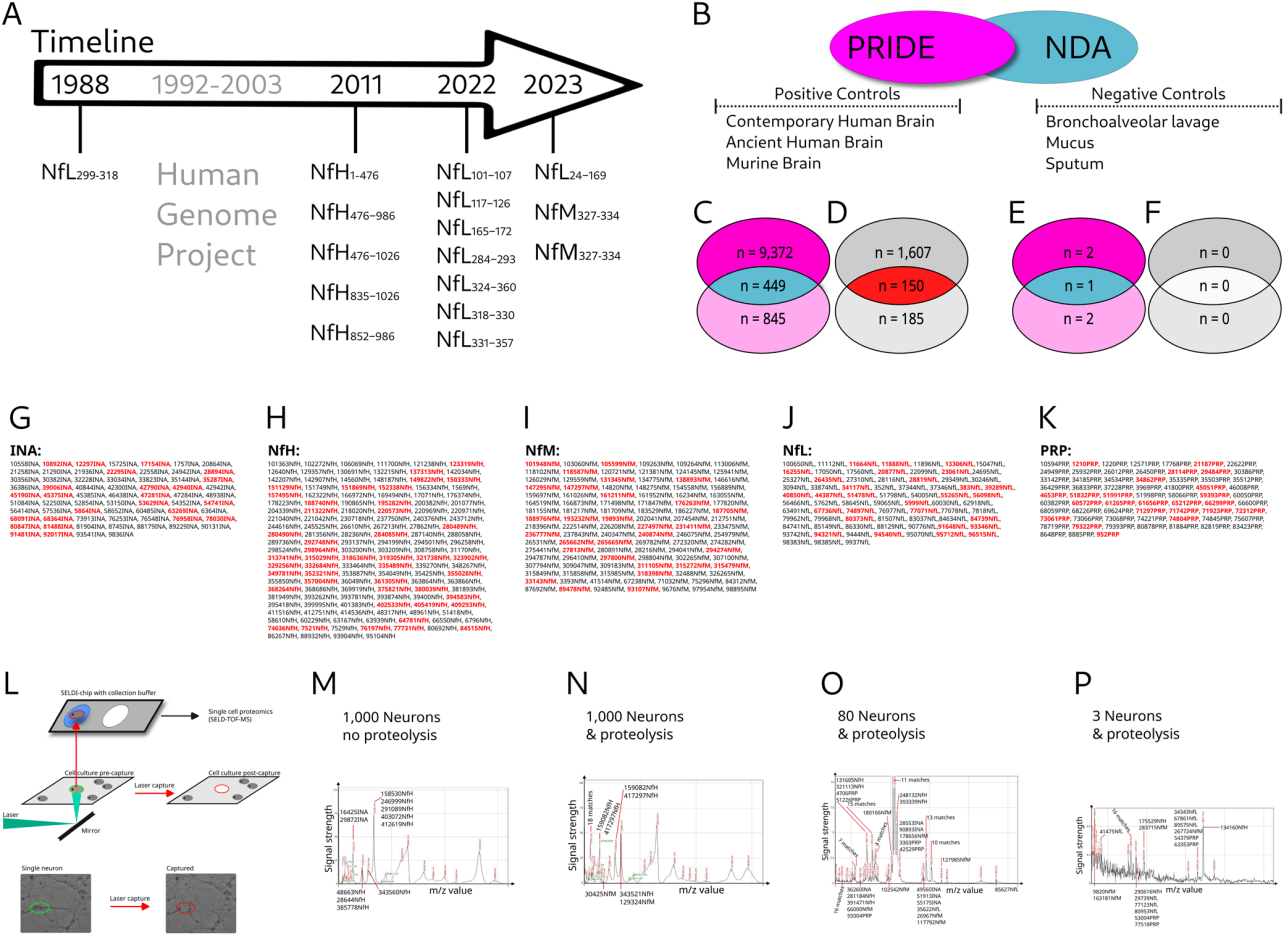
Validation of the NDA. (A) Validation from the literature (Table 5). Over the past 35 years, the sequence of 16 Nf cleavage products has been described. Most descriptions were made after the human genome project had become available. Consistent with the original publications in this figure, the amino acid positions are presented. (B) Validation through PRIDE repositories (Table 6). The PRIDE repository (coloured in magenta) data were matched with the NDA (coloured in blue). Matches were performed for three positive and three negative control datasets. (C) For the positive controls, there were 9372 matches of contemporary brain tissue (coloured in magenta) and 845 matches of ancient brain tissue (coloured in pink) with the NDA. Of these matches, 449 NDA peptides were present in both positive control databases (coloured in blue). (D) Reviewing the data from panel (C) for unique NDA peptides (panel H in Figure 4), the contemporary brain revealed 1607 matches (dark grey) and the ancient brain 185 matches (light grey). There were 150 unique NDA sequences present in both datasets. (E) For the negative controls, very few matches were found, (F) none of which were unique. (G) INA sequences validated from the PRIDE datasets matching both the contemporary and ancient human brain are shown. Unique NDA sequences are highlighted in red The Nf isoform ID refers to their corresponding unique identifier in the NDA. Likewise, these data are shown for (H) NfH, (I) NfM, (J) NfL, and (K) PRP. (L) Validation by single neuron proteomics. (M) Laser capture setup for single neuron proteomics. Individual cells are captured and collected in a buffer solution. The collected neurons are subjected to SELD‐TOF‐MS. The mass spectrum for SELDI‐TOF‐MS obtained is presented in panels (M–P). (M) spectra of methanol‐fixed neurons; (N) spectra of naive neurons. A comparison between the two reveals that naive neurons exhibit a greater number of absorption peaks, which are of slightly lower signal strength, presumably due to proteolysis. The *m*/*z* spectra are matched to the *m*/*z* values in the NDA. (O) Laser capture of 80 neurons yielded a diverse array of Nf isoform cleavage products. Peaks for unique Nf cleavage peptides were observed for NfM (3 peaks), NfH (2 peaks) and NfL (one peak). INA and PRP shared peaks with cleavage products of other Nf isoforms. (P) The minimum number of neurons providing detectable peaks required was three. The strongest signal originated from NfH (ID 134160NfH), followed by NfM (IDs 9820NfM, 163181NfM) and NfL (ID 41475NfL). The signal strength is shown on the *y*‐axis. Scales were 0%–100% (M, N), 10% (O), and 0.5% (P). Individual peaks were labelled with their *m*/*z* values.

Review of the literature identified 16 previously reported Nf cleavage products (Table 5). The first report of an NfL cleavage product sequence (Geisler et al. 1982) predated the Human Genome Project (Figure 7A). There is a 100% peptide sequence match with the then proposed 5 kDa NfL cleavage product, RAAKDEVSESRRLLKAKTLEIEAC (Geisler et al. 1982) and the NDA (ID 71540NfL, NfL_299‐318_). The literature also reveals five NfL cleavage products in Alzheimer disease (Budelier et al. 2022), two NfL cleavage products in spinal cord injury (Shaw et al. 2023), one NfL cleavage product and two NfM cleavage products from human induced pluripotent stem cells (Nezvedová et al. 2023) (iPSCs), and five NfH cleavage products identified from microdialysis human brain interstitial fluid in traumatic brain injury (Petzold et al. 2011).

**TABLE 4:** Pan‐Nf database containing common amino acid sequences shared among peptides from all Nf isoforms.

Pan‐Nf peptide	Amino acid sequence	Pan‐Nf peptide	Amino acid sequence
pan‐Nf_1_	LLNVKMALDIEIA	pan‐Nf_30_	YRKLLEG
pan‐Nf_2_	LLNVKMALDIEI	pan‐Nf_31_	ALDIEI
pan‐Nf_3_	LNVKMALDIEIA	pan‐Nf_32_	KLLEGE
pan‐Nf_4_	LLNVKMALDIE	pan‐Nf_33_	KMALDI
pan‐Nf_5_	LNVKMALDIEI	pan‐Nf_34_	LDIEIA
pan‐Nf_6_	NVKMALDIEIA	pan‐Nf_35_	LLEGEE
pan‐Nf_7_	LLNVKMALDI	pan‐Nf_36_	LLNVKM
pan‐Nf_8_	LNVKMALDIE	pan‐Nf_37_	LNDRFA
pan‐Nf_9_	NVKMALDIEI	pan‐Nf_38_	LNVKMA
pan‐Nf_10_	KMALDIEIA	pan‐Nf_39_	MALDIE
pan‐Nf_11_	LLNVKMALD	pan‐Nf_40_	NVKMAL
pan‐Nf_12_	LNVKMALDI	pan‐Nf_41_	RKLLEG
pan‐Nf_13_	NVKMALDIE	pan‐Nf_42_	YRKLLE
pan‐Nf_14_	YRKLLEGEE	pan‐Nf_43_	ALDIE
pan‐Nf_15_	KMALDIEI	pan‐Nf_44_	DIEIA
pan‐Nf_16_	LLNVKMAL	pan‐Nf_45_	KLLEG
pan‐Nf_17_	LNVKMALD	pan‐Nf_46_	KMALD
pan‐Nf_18_	MALDIEIA	pan‐Nf_47_	LDIEI
pan‐Nf_19_	NVKMALDI	pan‐Nf_48_	LEGEE
pan‐Nf_20_	RKLLEGEE	pan‐Nf_49_	LLEGE
pan‐Nf_21_	YRKLLEGE	pan‐Nf_50_	LLNVK
pan‐Nf_22_	ALDIEIA	pan‐Nf_51_	LNDRF
pan‐Nf_23_	KLLEGEE	pan‐Nf_52_	LNVKM
pan‐Nf_24_	KMALDIE	pan‐Nf_53_	MALDI
pan‐Nf_25_	LLNVKMA	pan‐Nf_54_	NDRFA
pan‐Nf_26_	LNVKMAL	pan‐Nf_55_	NVKMA
pan‐Nf_27_	MALDIEI	pan‐Nf_56_	RKLLE
pan‐Nf_28_	NVKMALD	pan‐Nf_57_	YRKLL
pan‐Nf_29_	RKLLEGE		

*Note:* Sequences are numbered and presented in descending order from 13 to 5 amino acids.

A review of PRIDE repositories provides both positive and negative control data (Figure 7B; Table 6). The validation study of positive controls revealed most matches from contemporary human brain tissue (PXD039808), followed by an archaeological human brain find (PXD014178, Figure 7C,D) and a murine brain (PRD000018). Conversely, for the negative controls (PXD039414), there was a notable scarcity of matches with the NDA (Figure 7E), and none with the bespoke unique NDA sequence (Figure 7F). For each of the PRIDE matches, the unique NDA sequences (as defined in Figure 4H) were highlighted (Figure 7G–K, coloured in red) and documented in the NDA.

**TABLE 5:** Validation of the NDA through comparison with published Nf isoform cleavage products.

Reference	Reported	Corresponding
	Cleavage product	NDA dataset
(Geisler et al. 1982)	NfL_299‐318_	ID 71540NfL
(Budelier et al. 2022)	NfL_101‐107_	ID 31180NfL
(Budelier et al. 2022)	NfL_117‐126_	ID 96153NfL
(Budelier et al. 2022)	NfL_165‐172_	ID 20556NfL
(Budelier et al. 2022)	NfL_284‐293_	ID 33351NfL
(Budelier et al. 2022)	NfL_324‐360_	ID 34575NfL
(Shaw et al. 2023)	NfL_318‐330_	ID 21567NfL
(Shaw et al. 2023)	NfL_331‐357_	ID 44228NfL
(Nezvedová et al. 2023)	NfL_24‐169_	ID 95822NfL
(Nezvedová et al. 2023)	NfM_327‐334_	ID 172256NfM
(Nezvedová et al. 2023)	NfM_383‐440_	ID 110837NfM
(Petzold et al. 2011)	NfH_1‐476_	ID 284507NfH
(Petzold et al. 2011)	NfH_476‐986_	ID 212532NfH
(Petzold et al. 2011)	NfH_476‐1026_	ID 212567NfH
(Petzold et al. 2011)	NfH_835‐1026_	ID 228836NfH
(Petzold et al. 2011)	NfH_852‐986_	ID 209506NfH

Likewise, single‐cell neuron proteomics unveiled precise SELDI‐TOF‐MS matches with the NDA (Figure 7L–P). Consistent with the in vitro experiments (Figure 3), the number of peaks increased with proteolysis (Figure 7M,N). These subtle peaks had been suppressed by the proprietary database, highlighting a role for the NDA as a lookup resource for low‐abundance Nf cleavage products in single‐cell experiments.

**TABLE 6:** Validation of the NDA through comparison with the PRIDE repositories (Basal et al. 2018; Petzold et al. 2020; Nezvedová et al. 2023).

Pride ID	Tissue	NDA sequences	
		All (*N*)	Unique (*N*)
Positive controls			
PXD039808	Human brain (formalin fixed)	9372	1607
PXD014178	Human brain (2600 years old)	848	185
PRD000018	Murine brain (development)	173	3
Negative controls			
PXD039414	Bronchoalveolar lavage	2	0
PXD039414	Mucus	2	0
PXD039414	Sputum	1	0

*Note:* The number (*N*) of matching cleavage products is shown.

### 3.4 Peptide Profile Features and Aggregation Potential

**FIGURE 8:**
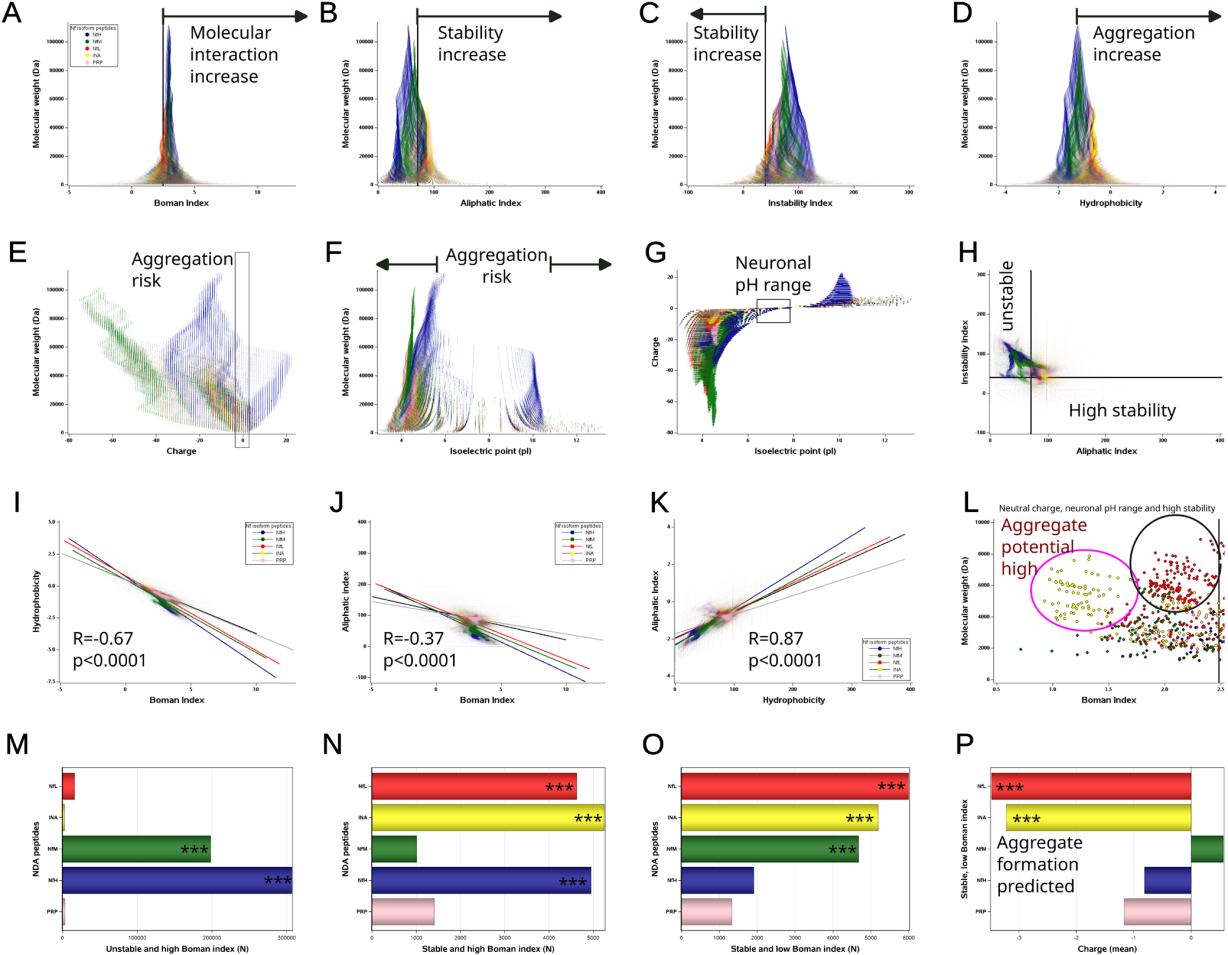
NDA peptide properties. (A) The Boman index surpasses the threshold (< 2.48, indicated by the vertical reference line) for full‐length Nf isoforms. Conversely, a low Boman index is exclusive to smaller NDA peptides (Molecular weight < 40 000 Da). A significantly higher proportion of NfL and INA cleavage products exhibit a low Boman index compared to the other Nf isoforms (Table 7). (B) An aliphatic index above 70 (vertical reference line) indicates increased stability. A significantly higher proportion of NfL, INA, and PRP cleavage products have a high aliphatic index when compared to NfH and NfM. (C) An instability index below 40 (vertical reference line) indicates increased stability. A significantly higher proportion of NfL and INA cleavage products are stable when compared to all other Nf isoforms. (D) Hydrophobicity is significantly increased in NfL, NDA, and PRP cleavage products compared to NfH and NfM, indicating a higher likelihood to aggregate in water‐based body fluids. (E) The charge of Nf isoform cleavage products is strongest for NfM compared to all other Nf isoforms (*p* < 0.0001), consistent with the charge repulsion theory, which states that the axonal diameter is predominantly governed by NfM. (F) The broad spread of the isoelectric points of the NDA peptides implies that most peptides are soluble at the physiological pH of 7.4. The inset (open oblong around charge 0) indicates the pool of NDA peptides at higher risk for aggregation based on charge. (G) NDA peptide precipitation is predicted for pH changes matching the peptide's pI. Experimentally, the pH range in neurons is from pH 6.6 to pH 7.9 (Chesler 2003) (highlighted in box). (H) A small cluster of mainly INA and NfL cleavage products is of predicted high thermodynamic stability. These peptides have an instability index below 40 (horizontal reference line) and an aliphatic index above 70 (vertical reference line). (I) The Boman index is significantly correlated with hydrophobicity. (J) The Boman index is significantly correlated with the aliphatic index. (K) There is a strong correlation between the aliphatic index and hydrophobicity. (L) For near‐neutral charge in the neuronal pH, there is a cluster of highly stable NDA peptides dominated by INA (open circle in magenta) and NfL (open circle in black). These peptides also have a low Boman index (vertical reference line) and are predicted to have a high potential for aggregation and precipitation. (M) A significantly higher proportion of NfH and NfM cleavage products are predicted to be unstable and of high molecular interaction potential and therefore unlikely candidates for aggregate formation. (N) The number of stable NDA peptides with a high Boman index is considerably lower compared to the unstable NDA peptide pool. (O) NfL and INA have a significantly higher number of NDA peptides that are of high thermodynamic stability and increased likelihood of aggregate formation. (P) A significantly more negative charge is found for NfL and INA cleavage products that are of high stability with a low Boman index, predicting a higher likelihood for aggregate formation. The analyses in panels (O) and (P) confirm on a statistical level the qualitative observations made in panel (L).

There are differences in the profile of peptide properties between Nf isoforms (Table 7). First, a Boman index of > 2.48, signifying a heightened level of molecular interaction (Karnati et al. 2022), is shown for over 90% of NfH and NfM peptides (Figure 8A), contrasting with a lower proportion for NfL and INA (Table 7). Second, the aliphatic index, indicating thermodynamic stability (Ikai 1980), applies to 92%–95% of INA and NfL peptides (Figure 8B). Third, the instability index (Gamage et al. 2019) also demonstrates heightened stability for INA and NfL peptides when compared to the other Nf isoforms (Figure 8C). Fourth, a higher hydrophobicity index, indicating a higher likelihood of protein aggregation (Barley et al. 2018; Valerio et al. 2004), also dominates the peptide profiles for INA, NfL and PRP when compared to NfH and NfM. In this profile plot, a cluster of INA, NfL and PRP peptides stands out (Figure 8D). Fifth, regarding the peptide profile charge, NfM stands out (Figure 8E), consistent with the dominant role NfM plays in the Nf heteropolymer to govern axonal diameter through charge repulsion (Khalil et al. 2024; Ding et al. 2024). Sixth, the likelihood of peptide aggregation increases markedly when the isoelectric point (pI) of a peptide approaches the surrounding pH, causing its net charge to approach zero (Figure 8F). This scenario applies to a small fraction of peptides (Figure 8G) where the in vivo brain extracellular fluid pH is 6.47–7.19 (Severinghaus and Astrup 1985; Timofeev et al. 2012). Seventh, combining both stability indices, Figure 8H shows that only a small fraction of NDA peptides are of high thermodynamic stability. Indices are inter‐correlated: Boman with hydrophobicity (*R* = −0.67, *p* < 0.0001, Figure 8I) and aliphatic (*R* = −0.37, *p* < 0.0001, Figure 8J), and hydrophobicity with aliphatic (*R* = 0.87, *p* < 0.0001, Figure 8K). Eighth, combining all peptide profile features associated with aggregate formation reveals a specific subset for INA and NFL (Figure 8L). Finally, the comparison of the peptide profile features across the NDA demonstrates that only NfM and NfH are likely to be metabolised easily because of their thermodynamic and molecular interaction features (Figure 8M). In contrast, INA and NFL dominate a small cluster of highly stable NDA peptides (Figure 8N,O), which, if taken together with all indices, harbour a statistically higher potential for aggregate formation in vivo (Figure 8P). The next section will focus on exploring these peptides in disease.

**TABLE 7:** Properties of the profile of Nf isoform cleavage products in the NDA.

	NfH	NfM	NfL	INA	PRP	Significance^a^
*N*	419 202	327 515	103 150	94 705	84 341	
Boman index	2.9 ± 0.4	3.0 ± 0.4	2.7 ± 0.6	2.7 ± 0.65	3.0 ± 0.7	*p* < 0.0001^b^
> 2.48	95.1%	92.6%	76.3%	76.2%	86.1%	
< 2.48	4.9%	7.4%	23.7%	23.8%	13.9%	
Aliphatic index	54.2 ± 19.7	64.2 ± 17.8	80.0 ± 18.8	89.2 ± 13.2	88.6 ± 15.1	*p* < 0.0001^c^
> 70	24.5%	34.6%	80.0%	94.7%	93.0%	
Instability index	82.2 ± 22.9	73.4 ± 18.3	58.9 ± 22.7	54.2 ± 16.6	60.4 ± 13.7	*p* < 0.0001^d^
< 40	2.5%	4.6%	11.9%	12.0%	4.0%	
Hydrophobicity	−1.3 ± 0.4	−1.2 ± 0.3	−0.8 ± 0.4	−0.7 ± 0.3	−0.8 ± 0.3	*p* < 0.0001^e^
Charge	−8.5 ± 13.7	−28.7 ± 22.2	−13.7 ± 13.1	−6.5 ± 5.6	−8.3 ± 6.7	*p* < 0.0001^f^
Isoelectric point	6.4 ± 2.3	4.9 ± 1.3	4.9 ± 1.4	5.4 ± 1.6	5.2 ± 1.6	*p* < 0.0001^g^

*Note:* Data are presented as mean ± standard deviation and rounded to the first digit. Numbers (N) and percentages (%) are also included. A Boman index greater than 2.48 indicates a higher probability of peptide interactions. An instability index of less than 40 and an aliphatic index greater than 70 and indicate enhanced thermodynamic stability.

^a^ The *p*‐value for the GLM is shown in the table. For subsequent individual group comparisons, *p*‐values are summarized in the table footnotes.

^b^ *p* < 0.0001 for all comparisons, except for NfH against NfM (*p* = 0.0084).

^c^ *p* < 0.0001 for all comparisons.

^d^ *p* < 0.0001 for all comparisons.

^e^ *p* < 0.0001 for all comparisons, except NfL against PRP (*p* = 0.0010).

^f^ *p* < 0.0001 for all comparisons, except NfH against PRP (*p* = 0.0047).

^g^ *p* < 0.0001 for all comparisons, except NfL against NfM (*p* = 0.25).

### 3.5 Disease Associations

Causal genes in rare neurodegenerative diseases have been linked to neurofilaments, with mutations in the NfL gene causing subforms of Charcot‐Marie‐Tooth Disease (CMT) (Mersiyanova et al. 2000; Higuchi and Takashima 2022; van Asperen et al. 2024), hereditary spastic paraplegia (HSP) (Mul et al. 2020), predisposing to amyotrophic lateral sclerosis (ALS) (van Asperen et al. 2024), spinal muscular atrophy (Ando et al. 2022) and Parkinson disease (PD) (Lavedan et al. 2002). These mutations alter the NDA profile.

#### 3.5.1 CMT Disease

**FIGURE 9:**
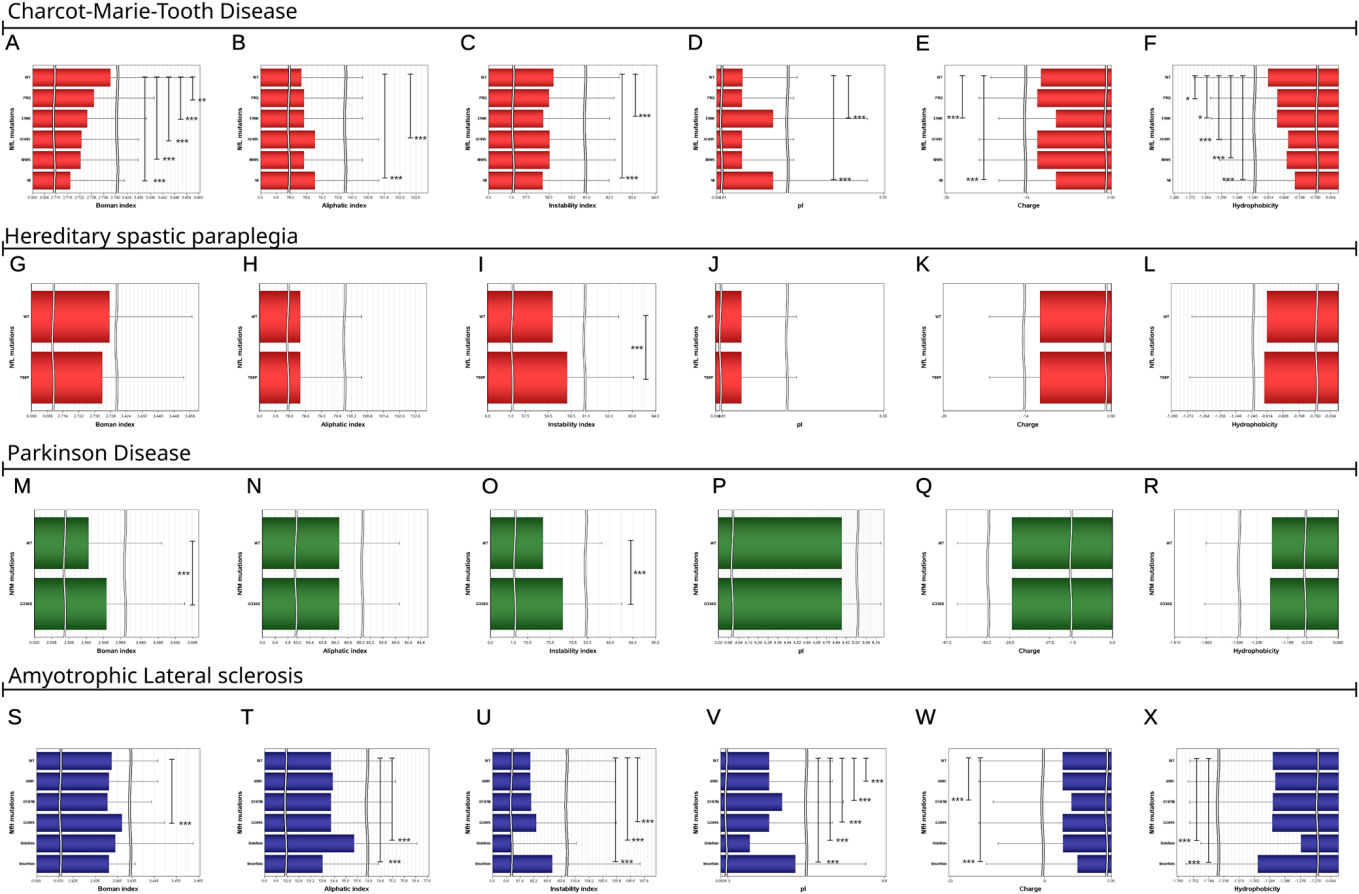
Neurological diseases caused by Nf mutations have alterations in the properties of the NDA peptide pool. (A) In CMT disease NfL mutations significantly reduced the Boman index (*F* value = 10.67, *p* < 0.0001), (B) increased the aliphatic index (*F* value = 10.67, *p* < 0.0001), (C) increased the instability index (*F* value = 10.67, *p* < 0.0001), (D) increased the pI (*F* value = 150.13, *p* < 0.0001), (E) lowered charge (*F* value = 39.62, *p* < 0.0001), (*F*) and reduced hydrophobicity (*F* value = 13.55, *p* < 0.0001). (G) In HSP there was no significant difference between the NfL T88P mutation and wild‐type for the Boman index, (H) the aliphatic index. (I) The instability index was significantly increased in the peptide pool of the NDA for the T88P mutation (*t* value = −6.08, *p* < 0.0001), but not for the (J) pI, (K) charge and (L) hydrophobicity. (M) In PD there is a significant increase of the Boman index with the NfM G236S mutation if compared to wild‐type (*t* value = −4.70, *p* < 0.0001), (N) the aliphatic index remains comparable, (O) the instability index increases significantly with the point mutation (*t* value = −18.28, *p* < 0.0001). There are no statistically significant changes for (P) the pI, (Q) charge, and (R) hydrophobicity. (S) In ALS only the NfH G249 point mutation significantly increased the Boman index compared to wild‐type (*F* value = 9.92, *p* < 0.0001), (T) increased (base pair deletion) and decreased (base pair insertion) the aliphatic index (*F* value = 275.62, *p* < 0.0001), (U) changed the instability index (*F* value = 103.92, *p* < 0.0001), (V) changed the pI (*F* value = 131.40, *p* < 0.0001), (W) changed charge (*F* value = 103.92, *p* < 0.0001), (X) and changed hydrophobicity (*F* value = 101.99, *p* < 0.0001).

The NfL gene mutations reported in CMT (Nefedova et al. 2023; Stone et al. 2019) significantly affected the Boman index of the corresponding NDA peptide profile if compared to wild‐type (Figure 9A); the aliphatic index (Figure 9B); the instability index (Figure 9C); the pI (Figure 9D); charge (Figure 9D) and hydrophobicity (Figure 9F). Taken together, the aggregation potential of the NfL peptide profile further increases with these mutations.

#### 3.5.2 Hereditary Spastic Paraplegia

In HSP, a founder effect was proposed for the T88P point mutation in the NfL gene (Mul et al. 2020). No statistically significant differences between this mutation and the wild‐type were found for the NDA peptide profiles (Figure 9G–L).

#### 3.5.3 Parkinson's Disease

In PD, it has been proposed that the NfM G336S mutation forms neuronal inclusions leading to the collapse of the cytoskeleton and neurodegeneration (Lavedan et al. 2002). This point mutation occurs in coil 2B of NfM, which is part of the *α*‐helic rod domain, essential for heteropolymer assembly. This argument is strengthened by findings from the NDA peptide profiles showing a significant loss of stability and heightened molecular interactions when compared to wild‐type (Figure 9M–R). There were 153 377 cleavage products unique to the NfM G336S mutation if compared to the NDA (available on figshare). This peptide pool could be used for the development of a diagnostic biomarker test for this mutation.

#### 3.5.4 Amyotrophic Lateral Sclerosis

An association with ALS has been reported for NfH gene point mutations, deletions, and insertions. Significant changes in the properties of the NDA peptide profile were observed, with deletions showing the opposite profile pattern to the insertions (Figure 9S–X). Whilst highly significant, the effect sizes of all findings are less than what was found for CMT, consistent with the difference between a causal gene mutation and a risk factor.

### 3.6 Aggregation‐Prone Regions

There is a diversity of aggregation‐prone regions (APRs) (Prabakaran et al. 2021) in the NDA. Comparative analysis reveals fewer APRs (*n* = 916, 0.2%) of the NfH fragments if compared to APR‐positive fragments from NfM (*n* = 226 620, 69.5%, *p* < 0.0001), NfL (*n* = 31 007, 30.3%, *p* < 0.0001), INA (*n* = 30 049, 31.9%, *p* < 0.0001) and PRP (*n* = 48 656, 58.2%, *p* < 0.0001). The odds ratio for the presence of APR‐positive fragments is notably elevated for NfM in comparison to NfH (1036.2688, 95% CI 970.8042–1106.1479, *p* < 0.0001), NfL (5.2414, 95% CI 5.1619–5.3220, *p* < 0.0001), INA (4.8447, 95% CI 4.7697–4.9209, *p* < 0.0001) and PRP (1.6338, 95% CI 1.6085–1.6596, *p* < 0.0001). These findings suggest a differential amyloidogenic seeding potential for Nf isoforms, an aspect that has yet to be systematically explored for its pathological relevance.

**FIGURE 10:**
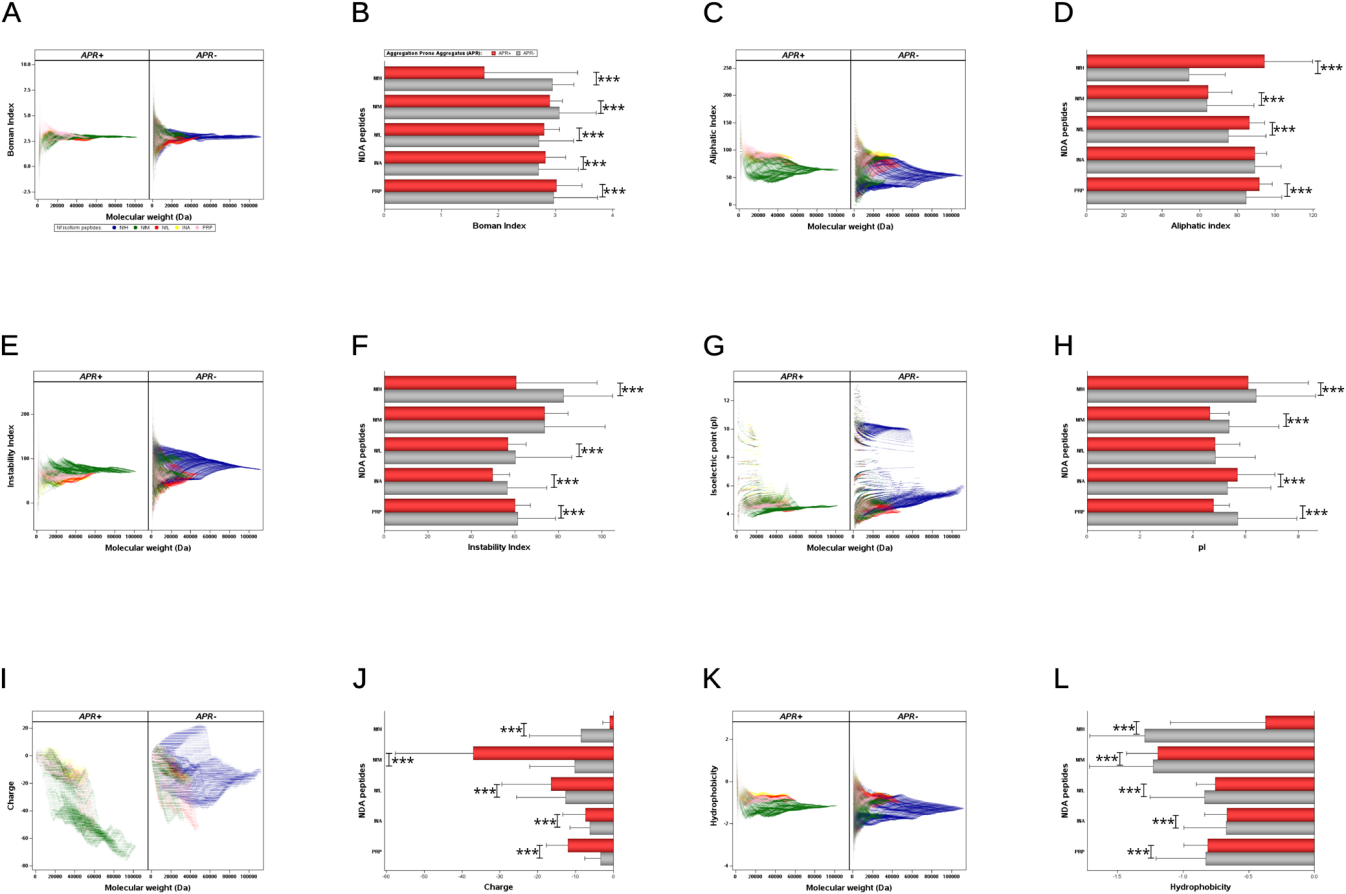
Aggregate prone regions (APR+) within the NDA exhibit heterogeneity. (A) The Boman index profile of the NDA indicates that APR+ regions vary across the molecular weight range. (B) In the APR+ NDA, the Boman index is significantly decreased for NfM and NfM, contrasting with increased levels in APR+ NDA for NfL, INA, and PRP. (C) The aliphatic index profile is higher in the APR+ NDA compared to the APR‐ NDA. (D) A significantly higher aliphatic index in APR+ NDA is observed for all Nf isoforms except INA. (E) The instability index profile is lower in APR+ NDA compared to APR‐ NDA. (F) The lower instability index in APR+ NDA reaches significance for NfH, NfL, and NfL, with an inverse relationship observed for PRP, and no difference for NfM. (G) The pI profile of the APR+ NDA is lower than that of the APR‐ NDA. (H) Significance of a lower pI in APR+ compared to APR‐ NDA is found for NfH, NfM, and INA, with an inverse relationship for PRP. (I) The charge profile of the APR+ NDA is lower than that of the APR‐ NDA. (J) Reduced charge in APR+ NDA is significant for all Nf isoforms except NfH. (K) The hydrophobicity profile of the APR+ NDA is reduced compared to APR‐ NDA. (L) Reduction in hydrophobicity in the APR+ NDA is significant for all Nf isoforms. Colour coding is consistent across Nf isoforms (inset in A) and APR+ NDA (red) and APR‐ NDA (grey, inset in B). *p* < 0.0001 = ***.

There are significant associations between APRs and the NDA peptide profile for the Boman index profile of APR‐positive NDA for the two largest Nf isoforms (NfH, NfM), which also harbour the highest proportion of KSP repeats (Khalil et al. 2024), which is inverse to the findings for the three shorter APR‐positive Nf isoforms (Figure 10A,B). The aliphatic index was significantly increased in APR‐positive NDA for all Nf isoforms except INA, compared to APR‐negative NDA (Figure 10C,D). In contrast, significant reductions were observed for the instability index, pI, charge and hydrophobicity (Figure 10E–K). Similarly, a significant reduction in pI was found in APR‐positive NDA compared to APR‐negative NDA for all Nf isoforms except NfL (Figure 10G,H). Charge was significantly reduced for all Nf isoforms except NfH (Figure 10I,J) as was hydrophobicity (Figure 10K,L). This heterogeneity of APRs suggests that different Nf isoform cleavage products may contribute to the nucleation and seeding of amyloid assembly in various chemical environments of the tissue.

### 3.7 Inhibition of Proteolysis

**FIGURE 11:**
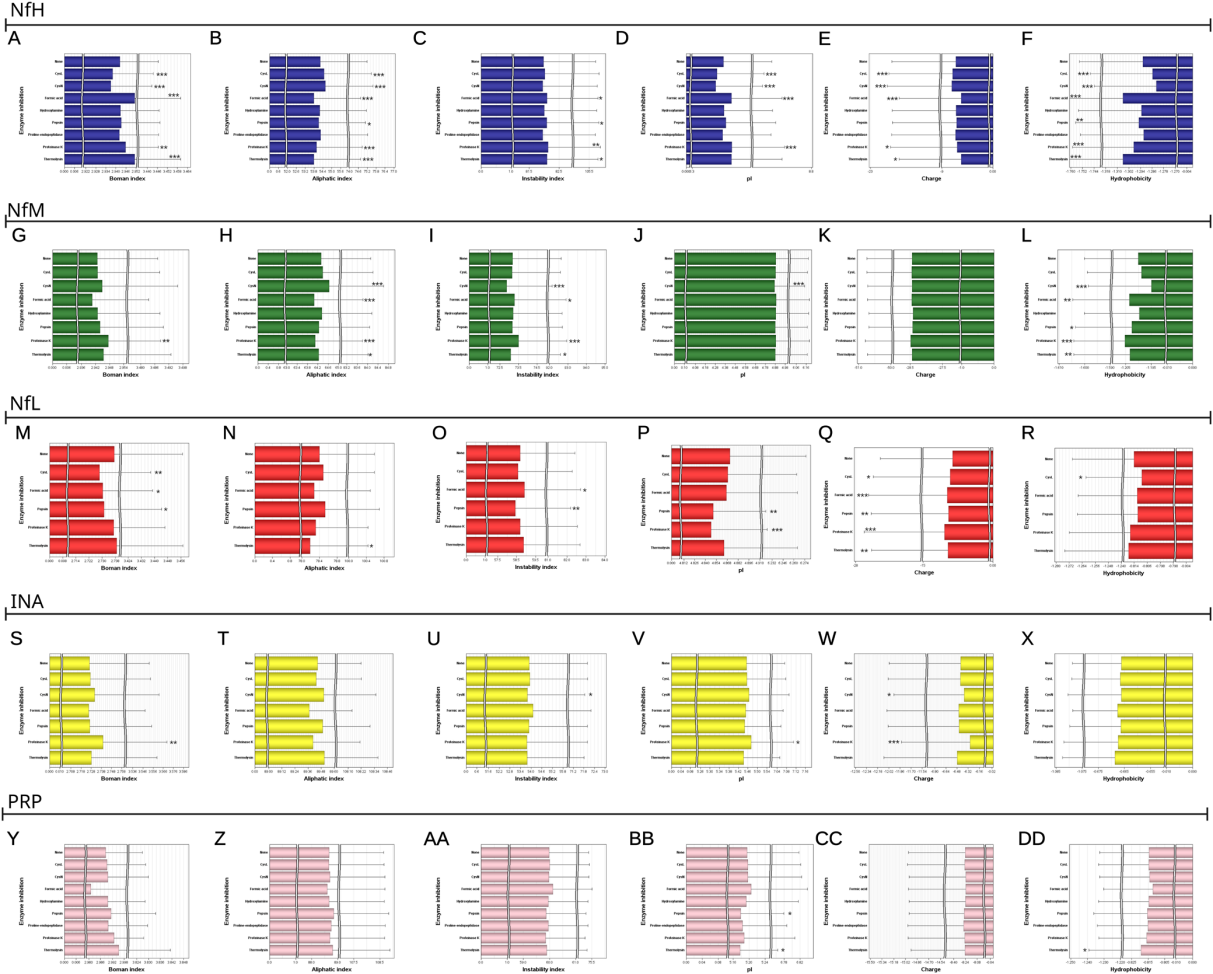
Selective enzyme inhibition changes the profile of NDA protein features. The selective cleavage sites for each of the Nf isoforms in the NDA are summarised in the last row of Table 1. These are the single cleavage sites only affected by one of the enzymes used to develop the NDA. The Figures show the changes in protein features following selective enzyme inhibition compared to the absence of enzyme inhibition (top row in each of the horizontal bar charts) enzyme inhibition (*p* < 0.0001 = ***, *p* < 0.001 = *, *p* < 0.01 = *). For NfH (in blue) there are multiple and significant changes for 30 of the protein features. (A) The Boman index, (B) aliphatic index, (C) instability index, (D) pI, (E) charge, and (F) hydrophobicity. For NfM (in green) changes are less extensive (*n* = 15) than what is seen for NfH. (G) The Boman index was only changed by inhibition of proteinase K. Three enzymes changed the (H) aliphatic index and (I) instability index. (J) The pI was changed only by inhibition of chymotrypsin‐N. (K) Charge remained unchanged. (L) Hydrophobicity was reduced by inhibition of five of the enzymes. For NfL (in red), significant changes were observed 14 times, for (M) the Boman index, (N) the aliphatic index, (O) the instability index, (P) the pI, (Q) charge, and (R) hydrophobicity. For INA (in yellow) only moderate changes were observed (*n* = 5) with enzyme inhibition. (S) The Boman index was increased by inhibition of proteinase K; (T) there were no significant changes in the aliphatic index, (U) the instability index was reduced by inhibition of chymotrypsin‐N, (V) the pI increased with inhibition of proteinkinase K, (W) charge was affected by both of these enzymes, and (X) there were no significant changes in INA hydrophobicity. For PRP (in pink), most protein features remained stable, notably (Y) the Boman index, (Z) the aliphatic index, (AA) the instability index; (BB) however, the pI changed with inhibition of pepsin and thermolysin. (CC) The charge of PRP remained stable. (DD) Reduction of hydrophobicity was calculated for inhibition of thermolysin.

Specific cleavage sites targeted by individual enzymes for each Nf isoform are listed in Table 1. For NfH, eight enzymes with one cleavage site were identified (Figure 11A–F), seven for NfM (Figure 11G–L), five for NfL (Figure 11M–R), six for INA (Figure 11S–X) and eight for PRP (Figure 11Y–DD). The impact of selective enzyme inhibition compared to no inhibition on key protein features is most pronounced for NfH (*n* = 30), followed by NfM (*n* = 15), NfL (*n* = 14), INA(*n* = 5) and PRP (*n* = 3). With few exceptions, the resulting changes in protein features show similar directional trends across different Nf isoforms (Table 8). Overall, the differential change in Nf isoform charge is most frequent. Identical directions of significant changes are observed for the aliphatic index (Figure 11B,H,N,T,Z) and hydrophobicity (Figure 11F,L,R,X,DD, Table 8). The NDA provides a resource for in silico screening of therapeutic targets to modify the peptide aggregation potential in Nf isoform‐driven neurodegeneration.

**TABLE 8:** The NDA profile for protein features that are changed by selective enzyme inhibition.

Enzyme inhibition	Boman index	Aliphatic index	Instability index	pI	Charge	Hydrophobicity
CysL	NfH↓NfM↓	NfH↑		NfH↓	NfH↓NfL↓	NfH↑
CysN	NfH↓	NfH↑NfM↑	NfM↓INA↓	NfH↓NfM↓	NfH↓INA↑	NfH↑NfM↑
Formic acid	NfH↑NfM↓	NfH↓NfM↓	NfH↑NfM↑NfL↑	NfH↑	NfH↑INA↓	NfH↓NfM↓
Hydroxylamine						
Pepsin	NfM↓	NfH↓	NfH↑NfL↓	INA↓PRP↓	NfL↓	NfH↓NfM↓
Proline‐endopeptidase						
Proteinkinase K	NfH↑NfM↑INA↑	NfH↓NfM↓	NfH↑NfM↑	NfH↑NfL↓INA↑	NfH↑NfL↓INA↑	NfH↓NfM↓
Thermolysin	NfH↑	NfH↓NfM↓NfL↓	NfH↑NfM↓	PRP↓	NfH↑NfL↓	NfH↓NfM↓

*Note:* A significant (see Figure 11) increase of a protein feature is indicated by an up‐arrow (↑) and a decrease by a down‐arrow (↓) for each of the Nf isoforms in the NDA.

## 4 Discussion

This study delivered a comprehensive resource for Nf biomarker research. The method for creating the NDA is generalisable to other proteins. Practical applications range from guiding affinity‐based biomarker assay development (Khalil et al. 2024; Shaw et al. 2023) and mass spectroscopy (Leckey et al. 2024) to experimental and clinical studies, including drug development (Figure 1). The NDA is rooted in mathematical combinatorics to accommodate for future expansion of the proteome (Tsuboyama et al. 2023; Uhlén et al. 2015). The NDA comprehensively and numerically catalogues proteolytic cleavage products, which are visualised through in vitro, in silico, and proteomic experiments. Three different and extensive validation studies were performed for the NDA from the literature data (Geisler et al. 1982; Budelier et al. 2022; Shaw et al. 2023; Nezvedová et al. 2023; Petzold et al. 2011), through openly available repositories (PXD039808, PXD014178, PRD000018, PXD039414) and in single‐cell proteomics. Fully annotated, these data are of value for targeted studies of selected Nf cleavage products already found to be relevant in neurodegenerative pathology (Bacioglu et al. 2016; Bjornevik et al. 2022; Leckey et al. 2024; Zamecnik et al. 2024). The novel described relationships between peptide profile metrics (Karnati et al. 2022; Ikai 1980; Gamage et al. 2019; Barley et al. 2018; Valerio et al. 2004), aggregation propensities (Prabakaran et al. 2021), and disease (Nefedova et al. 2023; Stone et al. 2019; Lavedan et al. 2002; van Asperen et al. 2024; Ando et al. 2022); links represent an important step forward in biomarker research because it overcomes recognised biases from the study of single proteins to the analysis of a profile of proteolytic breakdown products (Khalil et al. 2024; Shaw et al. 2023).

One limitation of the NDA is that our current understanding of cleavage sites remains incomplete (López‐Otín and Overall 2002; Tsuboyama et al. 2023; Uhlén et al. 2015). For example, the very recent discovery of NfL and INA degradation to be governed by the CRL3^gigaxonin^ ubiquitin ligase‐USP15 pathway (Park et al. 2023) has not been considered. Likewise, there is evidence for cleavage of Nf isoforms by calpains (Nixon et al. 1990; Stys and Jiang 2002). These enzymes are not included in PeptideCutter, which was used in this study. This limitation also applies to the development of a degradome atlas to any other protein. Therefore, an important strength of the NDA is to be rooted in mathematical combinatorics, which precisely describe the number of all possible cleavage products of any given degradome by $Z=\frac{\left(x+1\right)\times \left(x+2\right)}{2}-1$ (Figure 1). Another limitation is that the cleavage of proteins is likely to be compartmentalised and different between cell types. Future studies on this topic will find the NDA a valuable resource for interpretation of the mass spectroscopic readout of low‐abundance proteins in single‐cell proteomics. Another anatomical limitation stems from the choice of tissue at the interface of the central and peripheral nervous systems (Figure 3A). This decision reflects known differences in protein expression across various sites in the CNS (Hawrylycz et al. 2012).

The NDA will be found particularly useful for the identification of proteolytic Nf cleavage products that separate central from peripheral nervous system injuries (Petzold 2022; Khalil et al. 2024). A recognised limitation of earlier Nf studies is that Nf subunits, existing in both central and peripheral nervous systems, vary in their compartmentalisation (Petzold 2022; Khalil et al. 2024). Only very recently has it become possible to reliably quantify PRP (Keddie et al. 2023). For INA, such an assay still needs to be developed. The NDA empowers research focused on assay development with the selection of Nf subunit‐specific proteolytic cleavage products. Such selection can be refined by focusing on the thermodynamically most stable and soluble peptides, a prerequisite for any successful laboratory biomarker (Petzold 2022; Altmann et al. 2021; Khalil et al. 2024). In this context, the NDA can also aid in non‐invasive point‐of‐care test development. This includes the potential for detecting small Nf peptides in the urine which, as the NDA shows, often have properties favouring the urine's biochemical composition. Targeting small and specific Nf cleavage products from the NDA for monoclonal antibody generation is a promising approach.

The focus of the NDA was extended to the impact of mutations (Nefedova et al. 2023; Stone et al. 2019; Mul et al. 2020; van Asperen et al. 2024; Ando et al. 2022; Lavedan et al. 2002). This led to the creation of mutation‐specific databases, which are also made available for download. One finding was the significance of the profile of protein features, such as the Boman index (Boman 2003). The Boman index, underutilised in protein aggregation research, revealed that NfL, with a tendency for self‐assembly (Khalil et al. 2024; Zhou et al. 2022), produces over a hundredfold more cleavage products with a lower Boman index profile than NfH or PRP. Notably, pathogenic mutations in NfL (Bomont 2021; Mersiyanova et al. 2000; Higuchi and Takashima 2022; van Asperen et al. 2024) correlate with a significant shift to the left in the Boman index profile, suggesting increased aggregation propensity. Likewise, an association of INA with neurofilament inclusion body disease seems plausible based on the aggregation‐prone peptide profile.

The NDA also provides valuable insights into autoimmunity research (Ramanathan et al. 2023; Zamecnik et al. 2024). Initially linked with CJD and Kuru, its relevance has expanded to include a range of autoimmune disorders (Khalil et al. 2024). However, the pathogenic role of Nf autoantibodies remains inconclusive. With abundant matches in the NDA database, the majority of Nf autoantibodies might be an epiphenomenon (Sotelo et al. 1980; Ramanathan et al. 2023). Using the downloadable NDA algorithms, it is straightforward to expand this analysis to other protein autoimmune candidates.

The effects of inhibiting neurofilament (Nf) proteolysis have been previously documented (Nixon et al. 1990; Goldstein et al. 1987; Schlaepfer et al. 1985). Notably, increased phosphorylation has been shown to reduce proteolysis (Goldstein et al. 1987), which in turn raises the risk of Nf aggregate formation, a topic extensively reviewed recently (De Paoli et al. 2024). This review emphasised the potential value of NfH phosphorylation sites as biomarkers at the intersection of ageing and neurodegeneration. However, it also highlights the limitations of current assays in accurately quantifying phosphorylation changes (De Paoli et al. 2024). To address these challenges, the inclusion of NDA peptides containing serine residues, key phosphorylation sites for Nf isoforms, may be beneficial for advancing this area of research. Furthermore, environmental factors such as aluminium exposure and their relationship with Nf aggregation have been recognised as significant (Nixon et al. 1990). Nixon and colleagues suggested that aluminium may compete with phosphate for binding to the same amino acid residues, a mechanism warranting further investigation. Consequently, future studies examining the role of phosphorylation in Nf isoforms and its implications for disease (De Paoli et al. 2024) should consider simultaneously testing for aluminium and other neurotoxic substances.

In conclusion, the here‐presented systematic approach to Nf proteolysis provides an innovative and robust framework for addressing questions pertinent to protein aggregation and disease associations. The openly accessible datasets will be helpful for the selection of much‐needed specific and sensitive peptides that can be used for the development of affinity‐based and label‐free biomarker assays. Whilst the NDA is focused on Nf isoforms, the methodology is tailored to be generalisable to other proteins.

## Author Contributions

**Axel Petzold:** conceptualization, methodology, data curation, investigation, validation, formal analysis, visualization, writing – original draft, writing – review and editing, project administration.

## Conflicts of Interest

The author declares no conflicts of interest.

### Peer Review

The peer review history for this article is available at https://www.webofscience.com/api/gateway/wos/peer‐review/10.1111/jnc.70023.

## Data Availability

All datasets and codes have been uploaded to Figshare. The identifier is: https://doi.org/10.5522/04/25689378.v1 The files can be downloaded and used under the CC0 license.
